# Profiling of rhizosphere-associated microbial communities in North Alabama soils infested with varied levels of reniform nematodes

**DOI:** 10.3389/fpls.2025.1521579

**Published:** 2025-03-07

**Authors:** Sowndarya Karapareddy, Varsha C. Anche, Sowjanya R. Tamatamu, Madhusudhana R. Janga, Kathy Lawrence, Leopold M. Nyochembeng, Antonette Todd, Lloyd T. Walker, Venkateswara R. Sripathi

**Affiliations:** ^1^ College of Agricultural, Life & Natural Sciences, Alabama A&M University, Normal, AL, United States; ^2^ Institute of Genomics for Crop Abiotic Stress Tolerance, Department of Plant and Soil Science, Texas Tech University, Lubbock, TX, United States; ^3^ Department of Entomology and Plant Pathology, Auburn University, Auburn, AL, United States; ^4^ Department of Agriculture & Natural Resources, Delaware State University, Dover, DE, United States

**Keywords:** soil, rhizosphere, reniform nematode, infestation, Phyloseq, microbial diversity, bacterial and fungal communities

## Abstract

**Introduction:**

Plant roots, nematodes, and soil microorganisms have a complex interaction in the rhizosphere by exchanging or communicating through biomolecules or chemicals or signals. Some rhizospheric (including endophytic) microbes process such compounds via biogeochemical cycles to improve soil fertility, promote plant growth and development, and impart stress tolerance in plants. Some rhizospheric microbes can affect negatively on plant parasitic nematodes (PPNs) thus hindering the ability of nematodes in parasitizing the plant roots. Next-generation sequencing is one of the most widely used and cost-effective ways of determining the composition and diversity of microbiomes in such complex environmental samples.

**Methods:**

This study employed amplicon sequencing (Illumina/NextSeq) of 16S ribosomal RNA (16S rRNA) for bacteria and Internal Transcribed Spacer (ITS2) region for fungi to profile the soil microbiome in the rhizosphere of cotton grown in North Alabama. We isolated DNA (ZymoBIOMICS) from soil samples in triplicates from four representative locations of North Alabama. Based on the level of Reniform Nematode (RN) Infestation, these locations were classified as Group A-RN Not-Detected (ND), Group B-RN Low Infestation (LI), Group C-RN Medium Infestation (MI), and Group D-RN High Infestation (HI) and determined using sieving method and microscopic examination.

**Results and discussion:**

Our analyses identified 47,893 bacterial and 3,409 fungal Amplicon Sequence Variants (ASVs) across all groups. Among the bacterial ASVs, 12,758, 10,709, 12,153, and 11,360 unique ASVs were determined in Groups A, B, C, and D, respectively. While 663, 887, 480, and 326 unique fungal ASVs were identified in Groups A, B, C, and D, respectively. Also, the five most abundant rhizospheric bacterial genera identified were *Gaiella*, *Conexibacter*, *Bacillus*, *Blastococcus*, *Streptomyces*. Moreover, five abundant fungal genera belonging to *Fusarium, Aspergillus, Gibberella, Cladosporium, Lactera* were identified. The tight clustering of bacterial nodes in *Actinobacteria*, *Acidobacteria*, and *Proteobacteria* shows they are highly similar and often found together. On the other hand, the close association of *Ascomycota* and *Basidiomycota* suggesting that they have different ecological roles but occupy similar niches and contribute similar functions within the microbial community. The abundant microbial communities identified in this study had a role in nutrient recycling, soil health, plant resistance to some environmental stress and pests including nematodes, and biogeochemical cycles. Our findings will aid in broadening our understanding of how microbial communities interact with crops and nematodes in the rhizosphere, influencing plant growth and pest management.

## 1 Introduction

The rhizosphere, a critical zone of soil surrounding plant roots, serves as a dynamic interface for interactions between plants and a diverse array of microorganisms. These microorganisms, including bacteria, fungi, and archaea, play vital roles in enhancing plant growth, improving soil fertility, and promoting ecosystem stability. They are involved in various processes such as nutrient cycling, organic matter decomposition, and the regulation of plant stress responses (Bais et al., 2006; Zhalnina et al., 2022). The presence of beneficial microbes in the rhizosphere can improve plant health by enhancing nutrient uptake, providing protection against pathogens, and promoting plant growth through mechanisms like nitrogen fixation and phosphorus solubilization (Lugtenberg and Kamilova, 2009; Van der Heijden et al., 2015).

In addition to their direct benefits to plant health, rhizosphere microbes also interact with plant-parasitic nematodes (PPN’s), which are significant pests in agriculture. Nematodes, particularly those that feed on plant roots, cause substantial damage to crops by disrupting root function thereby affecting the plant growth that ultimately results in the yield loss. However, the rhizosphere is the niche to a wide variety of microorganisms that can influence nematode populations through several mechanisms. Beneficial bacteria and fungi in the rhizosphere can suppress nematode infestations by producing nematicidal compounds, competing for resources, or acting as biological control agents (Zhao et al., 2017; Singh et al., 2020).

Reniform nematodes (RN) is a devastating pest in agriculture due to their widespread distribution affecting several crop species and the ability to thrive in diverse soil conditions. Their infestations can significantly alter the microbial community structure within the rhizosphere, potentially leading to decreased microbial diversity and disrupted nutrient dynamics (van der Putten and Bakker, 2018). Studies have demonstrated that specific microbial taxa can improve plant health by suppressing nematode populations and enhancing nutrient availability (Latz et al., 2021). For instance, beneficial bacteria and fungi can establish symbiotic relationships with cotton roots, leading to improved nutrient uptake and overall plant vigor (Garbeva et al., 2004; Lugtenberg and Kamilova, 2009). Furthermore, these beneficial microbes in the rhizosphere can also produce bioactive compounds that directly inhibit hatching and development of nematodes (Prasad and De Vries, 2019). Identifying these microorganisms within cotton rhizospheres is crucial for developing innovative management strategies aimed at nematode control and soil nutrient enhancement, aiding in reducing the reliance on chemical pesticides (Hassan and Abo-Elyousr, 2019).

However, nematode infestations can significantly alter the structure and diversity of microbial communities in the rhizosphere (Liu et al., 2017; Naylor et al., 2021). Changes in microbial diversity, especially a reduction in beneficial bacteria and fungi, have been linked to increased nematode damage in crops such as cotton and soybean (Yuan et al., 2020a). Additionally, plant-parasitic nematodes (PPNs) can influence plant performance by altering root exudation patterns, which in turn modify the microbial composition of the rhizosphere and improve the availability of nitrogen (N) and phosphorus (P) to plants (Topalovic et al., 2020). Verschoor (2002) found that nematode feeding contributes to nutrient cycling through the excretion of ammonia (NH3), N defecation, and increased root exudation. Similarly, Xie et al. (2023) demonstrated that nematode infestations in rice altered microbial populations enhancing nutrient cycling, particularly by increasing nitrogen-fixing bacteria that support plant growth. Wang et al. (2022) reported that nematode feeding on wheat roots shifted microbial communities, favoring fungi that contribute to organic matter decomposition, thus enhancing soil nutrient availability. In another study, Patel et al. (2024) showed that nematode-induced changes in microbial diversity helped plants by promoting the activity of specific microbes involved in phosphorus cycling, supporting plant growth under nutrient-limited conditions. Increased nematode presence often correlates with a decline in beneficial microbes, disrupting the ecological balance and negatively impacting soil health (Zhao et al., 2018; Bhattacharyya and Jha, 2012). Therefore, understanding the interplay between nematodes and microbial communities is essential for fostering sustainable agricultural practices.

The interactions between nematodes and soil microorganisms are multifaceted, encompassing competition, predation, and mutualism (Cai et al., 2023). Beneficial microbes can suppress nematode populations through antagonistic mechanisms, while nematodes may alter microbial community dynamics by changing resource availability (Gomez et al., 2019). Recent studies have emphasized the role of certain bacterial phyla, such as *Proteobacteria*, *Firmicutes*, and *Actinobacteria*, in suppressing nematode populations and promoting plant health. For example, *Proteobacteria* has been shown to produce metabolites that can inhibit nematode development, while *Firmicutes* and *Actinobacteria* contribute to enhanced plant nutrient uptake and nematode resistance (Li et al., 2022; Zhang et al., 2020). A study proposed by Naylor and Gurevitch (2021) that nematode feeding can change the composition of these microbial communities, often favoring *Ascomycota* and *Basidiomycota*, which can either help control nematode populations or shift microbial balance in ways that may reduce plant vitality. Additionally, nematodes themselves can modulate the structure of these microbial communities, causing a decline in beneficial microbes, such as those from the *Proteobacteria*, which can have cascading effects on soil health and plant resilience (Shang and Wang, 2022). Some studies have reported shifts in microbial diversity and composition in response to nematode presence, with certain taxa thriving while others diminish (De Vries and Shade, 2013). Investigating these dynamics across varying infestation levels can provide insights into how nematodes impact microbial communities and their function.

Furthermore, understanding microbial shifts in response to nematode infestations can lead to the development of targeted microbial inoculants or soil amendments that enhance beneficial microbial populations (Luo et al., 2022). These strategies offer sustainable alternatives to chemical controls, promoting long-term soil health and resilience in cotton species (Wu et al., 2019). By fostering beneficial microbial communities, it may be possible to mitigate the adverse effects of nematodes on cotton production and improve overall soil health. Advancements in molecular techniques, specifically 16S rRNA and ITS2 sequencing, have revolutionized the study of rhizosphere microbial communities. The 16S rRNA gene serves as a universal marker for bacterial identification, while the ITS2 region is widely used for characterizing fungal diversity (Ranjan et al., 2020). Together, these sequencing techniques provide a comprehensive view of the microbiome, revealing complex interactions that can influence plant health and stress responses. Incorporating R and the Phyloseq package into data analysis allows for robust profiling of microbial communities derived from sequencing studies. Phyloseq offers an efficient framework for handling and visualizing complex ecological data, enabling in-depth analysis of microbial diversity, community composition, and potential functional roles within the rhizosphere (McGuire and Triplett, 2009). This approach is particularly useful for examining the influence of RN’s on microbial dynamics in cotton soils, facilitating a deeper understanding of how these interactions impact plant health and productivity.

This study aims to profile the rhizosphere microbiome of cotton soils infested with RN’s across various infestation levels in North Alabama. By employing 16S rRNA and ITS2 sequencing, combined with analyses in Phyloseq, we seek to explore the intricate relationships between nematodes and microbial communities. This investigation will help identify key microbial taxa associated with different infestation levels of RN, offering insights into potential indicators of soil health and crop resilience.

## 2 Materials and methods

### 2.1 Field site selection and sample collection

The experimental design of this study primarily aimed at profiling rhizospheric microbial communities of morphometrically classified Reniform Nematode infestation levels (Nyaku et al., 2013a, b) in selected locations of North Alabama. Alabama climate is humid and subtropical geographically spread between the Gulf of Mexico at the Southern end and Appalachian Mountains at North-eastern proximity. The climatic conditions in North Alabama are uniform across these soil sample collected locations without considering the micro-climatic factors. As climatic factors and agricultural practices are relatively uniform, slight differences in soil types and the effects of soil properties on microbiome were not emphasized in our study. The soils in Jackson, Lauderdale, Madison, and Limestone counties are primarily derived from limestone and sandstone. In the selected locations, cotton is grown as monocrop or dual crop with soybean. These soils include Decatur, Dewey, Bodine, Fullerton, Madison, Pacolet, and Cecil series, featuring textures like clayey with silt loam and sandy loam surfaces (Alabama Cooperative Extension System, 2020). Sampling locations and GPS-determined coordinates of four selected sites were outlined in **Supplementary Figure 1**, **Table 1**, respectively.

**Table 1:** Geographic locations and coordinates of four counties of North Alabama.

RN Infestation Level/Groups	County Name	Location of the sample collected	Latitude	Longitude	Altitude (m)
ND-A	Jackson	Scottsboro	34.621466	-86.170195	196
LI-B	Lauderdale	Florence	34.789377	-87.746495	148
MI-C	Madison	Huntsville	34.784381	-86.505875	204
HI-D	Limestone	Belle Mina	34.661774	-86.879342	179

RN, Reniform Nematode; ND, Not-Detected; LI, Low Infestation; MI, Medium Infestation; HI, High Infestation.

Soil samples were collected from four counties of North Alabama, USA, based on RN infestation levels: Group A - RN Not-Detected (ND), Group B - RN Low Infestation (LI), Group C - RN Medium Infestation (MI), and Group D - RN High Infestation (HI) across Jackson (ND), Lauderdale (LI), Madison (MI), and Limestone (HI), respectively. Varied levels of RN infestation and their distribution in North Alabama were determined based on our previous studies such as morphometric and DNA-based (18S and ITS) marker analyses (Nyaku et al., 2023, 2016, 2013a, 2013b), and also as reported in similar agricultural studies (Roe and Owens, 2017; Thomas et al., 2019). The infestation levels of RN in the soil samples were determined using the sieving method, where soil samples were passed through a series of sieves to isolate nematodes. First, 25 ml of soil solution with nematodes was collected from 100g of soil using a sieve method. Then, 1 ml of soil solution was aliquoted and used to count the number of nematodes under the microscope to assess morphometrically and categorize them across various infestation levels. Where, ND = 0 RN detected, LI = <2,000 RN detected, MI = 2,000-5,000 RN detected, and HI = > 5,000 RN detected. This method ensures reliable classification of the RN infestation levels, which were based on previous studies and established protocols for nematode extraction and quantification (Eisenback and Triantaphyllou, 1991; Siddiqi, 2000).

The soil sampling and collection procedures used were meticulously adhered to the Alabama Cooperative Extension System protocol (Celleti & Potter, 2006) to ensure the highest data quality for our study. Recent guidelines on soil sample handling and preservation (de la Fuente et al., 2021) were followed to minimize contamination risks and maintain microbial integrity. Rhizospheric soils were collected at a depth of approximately 10-20 cm and <12 cm from the crop using soil auger as recommended (Smith & Lee, 2023). Plant debris (including roots), stones, and other impurities were removed during the collection process. Triplicate samples of 500g for each location were collected and placed in sterile zip-lock bags. Then these samples were transported in a dark cooler with ice and stored at 4^0^C until further processing (Li et al., 2023). Twelve samples collected (four counties and three replicates) were processed for nematode isolation and quantification and genomic DNA isolation. Same sample source has been used to quantify and characterize reniform nematodes for determining their levels of infestation and to isolate the DNA with higher integrity.

Nematodes were collected from the soils of Jackson, Lauderdale, Madison, and Limestone counties, morphometric measurements were made on male and female nematodes using an Olympus microscope (Olympus Optical Co. Ltd, Japan). The morphometric variables used for accurately determining the RN and their distribution in Alabama were body length, stylet length, position of vulva, spicule length, length of hyaline portion of tail, position of dorsal oesophageal gland orifice, position of excretory pore, maximum width, esophageal length and anal width. Prior to DNA extraction, the soil samples were thoroughly mixed to ensure uniformity and consistency. This step is crucial for ensuring reliable and consistent results in downstream microbiome analysis (Garcia-Sanchez et al., 2020). About 500mg of soil was measured in triplicates in 2-ml sterile microcentrifuge tubes for DNA isolation (4 x 3 = 12 samples).

### 2.2 Soil DNA extraction, library preparation, and sequencing

DNA was extracted from 12 soil samples using the ZymoBIOMICS-96 MagBead DNA Kit (Zymo Research, Irvine, CA), according to the manufacturer’s instructions. The elution volume of DNA is 50 ul. The quantity and quality of the isolated DNA were assessed (Sambrook and Russell, 2001) using Nanodrop 1000 Spectrophotometer (Thermo Fisher Scientific, 2023), Qubit 1X dsDNA Broad Range Assay Kit (Invitrogen, 2016), and Agarose Gel Electrophoresis (Wilson, 2020), respectively. Bacterial 16S rRNA gene sequencing was conducted using the Quick-16S NGS Library Prep Kit (Zymo Research, Irvine, CA), specifically targeting the V3–V4 region of the 16S rRNA gene. Amplification was performed with designated bacterial 16S primers, adhering to the following PCR protocol: an initial denaturation step at 95°C for 3 minutes, followed by 25 cycles of 95°C for 30 seconds, 55°C for 30 seconds, and 72°C for 30 seconds, concluded with a final extension at 72°C for 5 minutes. Each sample underwent triplicate processing to enhance reproducibility (Mardis, 2008). For fungal analysis, ITS2 gene sequencing was similarly executed using the Quick-16S NGS Library Prep Kit, replacing the 16S primers with custom ITS2 primers from the Microbiome Sequencing ITS2 Primer Set. The PCR conditions for the ITS2 amplification included an initial denaturation at 95°C for 3 minutes, followed by 30 cycles of 95°C for 30 seconds, 55°C for 30 seconds, and 72°C for 30 seconds, and a final extension at 72°C for 5 minutes (White et al., 1990).

To minimize PCR chimera formation, real-time PCR monitoring was employed during library preparation for each sample. The resulting PCR products were quantified using qPCR fluorescence readings and pooled based on equal molarity. The pooled library underwent purification using the Select-a-Size DNA Clean and Concentrator (Zymo Research, Irvine, CA) and was quantified using TapeStation (Agilent Technologies, Santa Clara, CA) and Qubit 1X dsDNA High-Sensitivity Assay Kits (Thermo Fisher Scientific, Waltham, WA) (Parker et al., 2016). ZymoBIOMICS Microbial Community DNA Standards (Zymo Research, Irvine, CA) served as positive controls for each DNA extraction and targeted library preparation. Additionally, negative controls, including blank extraction and library preparation controls, were incorporated to assess the quality and potential contamination during these processes (Kozich et al., 2013). In total, 12 libraries were sequenced on the Illumina NextSeq 2000 using a p1 (cat 20075294) reagent kit (600 cycles), with a 30% PhiX spike-in control included for sequencing (Illumina, 2019).

### 2.3 Bioinformatics and statistical analysis

Bioinformatics analyses were conducted to process and analyze the sequence data, starting with the improvement of read quality (Bolger et al., 2014; Chen et al., 2022). Then, the reads were paired together and assembled into genetic sequences, which were subsequently compared to reference genomes for organism identification (Li et al., 2021; Bushnell et al., 2017). The raw reads from amplicon sequencing data (16S rRNA and ITS2) were processed using the Divisive Amplicon Denoising Algorithm 2 (DADA2) pipeline in R (v4.3.2), following the procedure outlined by Callahan et al. (2016a, b). Data were then statistically analyzed with Phyloseq (v1.46.0) to create a data matrix and examine microbiome differences across and within samples. The DADA2 workflow involves quality filtering and trimming, de-replication, sequence table construction, chimera removal, taxonomy assignment, and phylogenetic tree construction. In the first step, forward reads were truncated at position 300 and reverse reads at position 200 for the 16S rRNA dataset, while for the ITS2 dataset, forward reads were truncated at position 180 and reverse reads at position 250. After being filtered by DADA2, the reads were grouped into distinct Amplicon Sequence Variants (ASVs) and aligned using the DECIPHER R package (Wright, 2015). Then, dereplication was performed to eliminate redundancy and infer ASVs without applying any arbitrary threshold, allowing for the detection of variants that differ by as little as a single nucleotide. Next, chimeras were subsequently removed using the “removeBimeraDenovo” command. Subsequently, taxonomy was assigned using the naive Bayesian classifier, employing the Ribosomal Database Project (RDP) v19 training set for 16S rRNA data (Wang et al., 2007; Cole et al., 2014) and the UNITE database v9.0 (Abarenkov et al., 2023) for ITS2 data and the phylogenetic tree was constructed with the Phangorn R package (Schliep, 2011). Finally, a Phyloseq object was used to import all the data to carry out alpha diversity, beta diversity, relative abundance with composition barplots, differential abundance analysis, heatmap, and network analyses.

Subsequently, R (v4.3.2) was used to conduct statistical analyses and visualizations using Phyloseq (v1.46.0) and additional packages such as VennDiagram (Chen and Boutros, 2011), UpsetR (Conway et al., 2017), ggplot2 (Wickham, 2016), gridExtra (Auguie, 2017), tidyverse (Wickham et al., 2019), vegan (Oksanen et al., 2020), ggpubr (Kassambara, 2020), reshape2 (Wickham, 2007), plotly (Sievert, 2020), microbiomeutilities (Lahti and Shetty, 2017), ampvis2 (Andersen et al., 2018), and microbiotaProcess (Xu et al., 2021). In short, a Phyloseq object was used to import all the data (McMurdie and Holmes, 2013). The “alpha” function from the Microbiome package (Lahti and Shetty, 2017) was used to compute alpha diversity. Rarefaction curves of the Shannon bacterial ASVs were computed using the Vegan package. Using the methods in the Phyloseq package, beta diversity was analyzed by Weighted Unifrac Bray-Curti’s distance (Lozupone et al., 2011) calculations and plotting and visualization with the Phyloseq package.

Relative abundance of the taxa was determined and agglomerated at the phylum, family, and genus levels using the Phyloseq. Venn diagrams were created and UpsetR packages were used to illustrate the number of ASVs unique and common among different communities (Chen and Boutros, 2011). The core bacterial microbiome of soil samples was calculated based on relative abundance using “Microbiome analyst” 2.0 (Chong et al., 2023). Differential abundance of microbial groups was assessed using DESeq2 (Love et al., 2014), with biomarker characteristics identified based on significant treatment-related changes (*p* < 0.05) and an effect size > ± 1 (log2FoldChange > ± 1). All analyses were considered statistically significant at a p-value of less than or equal to 0.01, except for DESeq2 analysis (Love et al., 2014). Finally, network plots were generated using the Phyloseq package in R (McMurdie and Holmes, 2013), which involved creating an object from the microbiome data, followed by the application of the igraph package (Csardi and Nepusz, 2006) to visualize relationships among taxa based on co-occurrence patterns.

## 3 Results

### 3.1 Microbiome richness and diversity

A total of 7,485,810 (7.49 million) and 8,088,548 (8.09 million) raw reads (R1+R2) of 16S rRNA and ITS2, respectively were obtained from soil samples collected from four counties of North Alabama in triplicates. After quality control and trimming using DADA2, we retained 7,204,800 (7.20 million) bacterial and 7,759,796 (7.76 million) fungal high quality sequences. The final unique sequences collected after trimming, dereplicating, filtering chimeric regions, and size selection for bacterial (**Table 2**) and fungal (**Table 3**) sequences were presented. Our taxonomic assignment from the DADA2 pipeline revealed novel and intriguing insights when we compared our samples against the RDP v19 training set for 16S rRNA and UNITE database for ITS data analyses.

**Table 2:** Read summary table for the soil samples from 16S rRNA Sequencing.

RN Infestation Level/Groups	County Name	rawseqs(R1+R2)	trimmed_seqs(R1+R2)	chimera_seqs	seqs(after_size_filtration)	final_unique_seqs
ND-A	Jackson	2288000	2201756	84424	813571	12325
LI-B	Lauderdale	1528770	1471846	44899	483396	10344
MI-C	Madison	1611864	1550944	50517	488691	11014
HI-D	Limestone	2057176	1980254	46635	820781	12232

RN, Reniform Nematode; ND, Not-Detected; LI, Low Infestation; MI, Medium Infestation; HI, High Infestation.

**Table 3:** Read summary table for the soil samples from ITS2 Sequencing.

RN Infestation Level/Groups	County Name	rawseqs(R1+R2)	trimmed_seqs(R1+R2)	chimera_seqs	seqs(after_size_filtration)	final_unique_seqs
ND-A	Jackson	2055524	1971520	24407	942064	2446
LI-B	Lauderdale	1909194	1831098	26534	866174	3105
MI-C	Madison	1668612	1601018	43696	731102	1556
HI-D	Limestone	2455218	2356160	52945	1110584	747

RN, Reniform Nematode; ND, Not-Detected; LI, Low Infestation; MI, Medium Infestation; HI, High Infestation.

### 3.2 Alpha diversity

**Figure 1:**
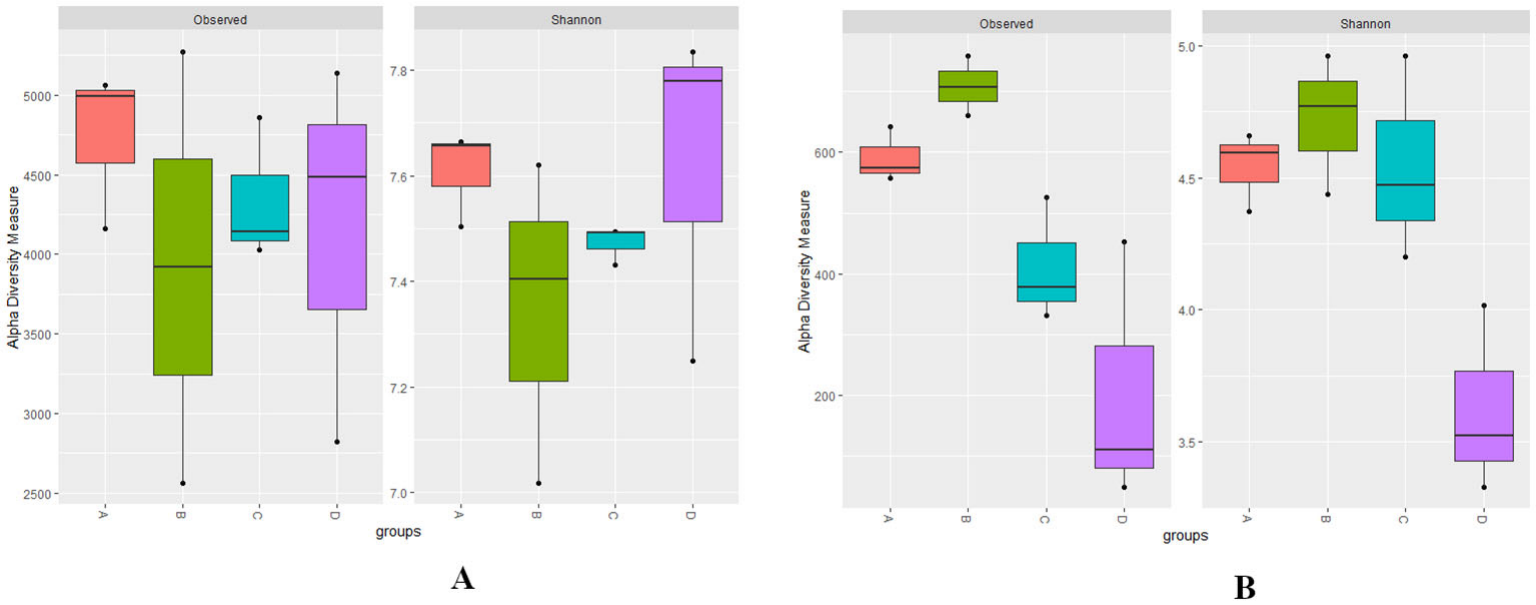
**(A, B)** Boxplots representing the bacterial observed, bacterial Shannon, fungal observed, and fungal Shannon indices at different levels of RN Infestation (*p*<0.01, Kruskal–Wallis test). Group A-RN Not-Detected, Group B-RN Low Infestation, Group C-RN Medium Infestation, Group D-RN High Infestation.

Metrics of alpha diversity are employed to assess the richness and evenness of a sample’s microbial community at various levels of RN infestation with Kruskal–Wallis test (*p* < 0.01), providing insights into microbial community composition (Lundberg et al., 2020a; Allen and Banfield, 2021). The observed species revealed higher bacterial richness in Group A indicating greater species diversity, while a higher bacterial richness and evenness (Shannon) was identified in Group D with *p* < 0.01 reflecting a more even distribution of species within the microbial community compared to the other groups (**Figures 1A**). Whereas, the fungal communities in Group A exhibited higher richness and evenness (Shannon index) and higher richness with observed species in Group B with a statistically significance (*p* < 0.01). However, in Group D, the richness for observed species was lower when compared with the Shannon index for richness and evenness (**Figures 1B**).

**Figure 2:**
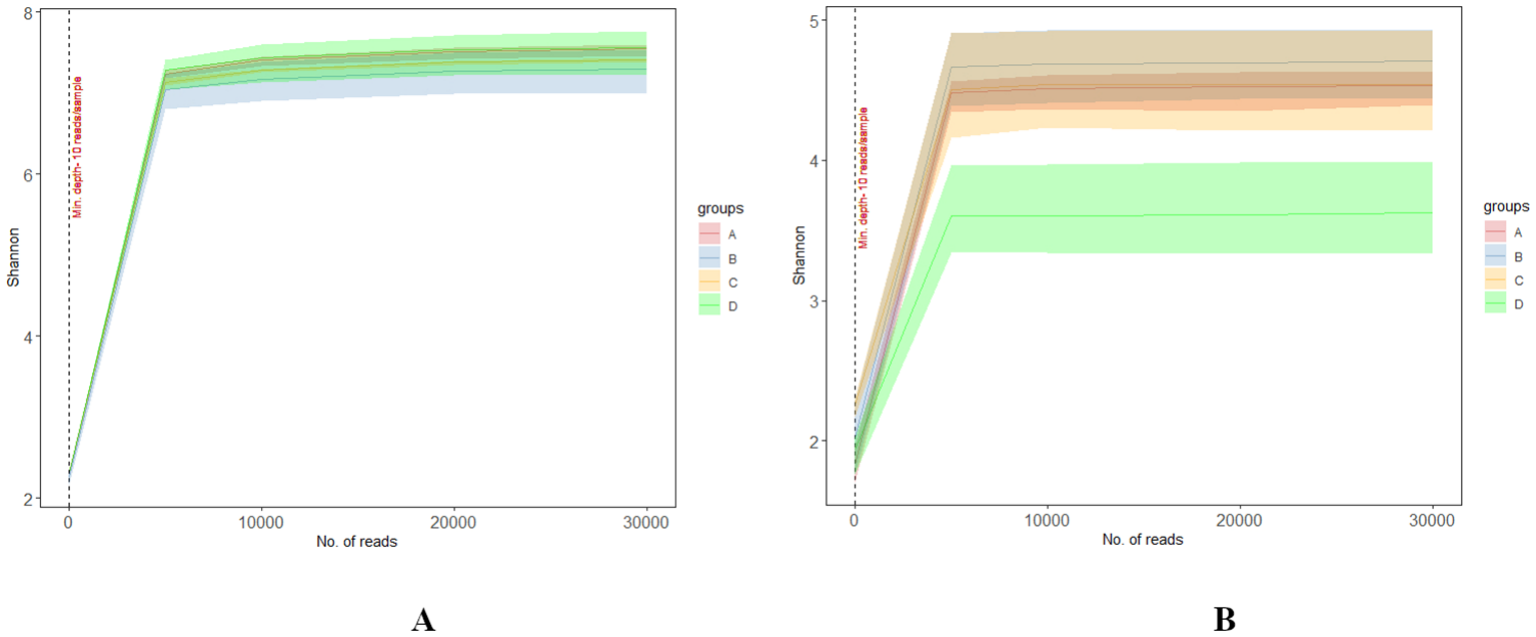
**(A, B)** Rarefaction curves illustrate the Shannon diversity indices of bacterial and fungal communities at different levels of RN Infestation with statistical significance (*p* < 0.01). Group A-RN Not-Detected, Group B- RN Low Infestation, Group C- RN Medium Infestation, Group D- RN High Infestation.

Shannon rarefaction curves indicated similar trends in microbial diversity across various levels of RN infestation for both bacterial and fungal communities (**Figures 2A, B**). Specifically, the bacterial communities in Group D exhibited the highest microbial diversity, with values ranging from 7.34 to 7.67, while Group B showed the lowest diversity, ranging from 6.78 to 7.27 (**Figure 2A**). In contrast, the fungal communities revealed that Group A had the highest microbial diversity, ranging from 4.28 to 4.85, whereas Group D exhibited the lowest diversity, with values ranging from 3.17 to 3.94 (**Figure 2B**). Notably, after reaching 30,000 sequences, the Shannon index plateaued at the 97% similarity threshold (α = 0.03), indicating that sufficient sequences were obtained to meet the sequencing requirements (Olesen and Simmelsgaard, 2019). The Shannon rarefaction curves for both bacterial and fungal samples (**Figures 2A, B**) illustrated that the RN infestation curves increased linearly before stabilizing suggesting that the sequencing data was reliable for further investigation (Chao et al., 2014).

### 3.3 Beta diversity

**Figure 3:**
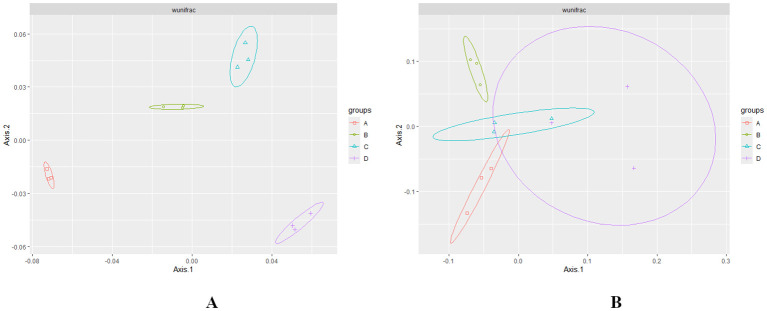
**(A, B)** Principal Coordinate Analysis (PCoA) plot based on Bray-Curtis weighted uniFrac showing the distance in the bacterial and fungal communities at different levels of RN Infestation. Significance was tested using PERMANOVA test (*p* < 0.01). Group A-RN Not-Detected, Group B- RN Low Infestation, Group C- RN Medium Infestation, Group D- RN High Infestation.

PERMANOVA analysis of weighted UniFrac distances revealed significant differences (*p* < 0.01) in microbial composition at various levels of RN infestation (Gauthier et al., 2022). The beta diversity or principal coordinate analysis (PCoA) plot, based on weighted UniFrac distances, demonstrated that bacterial groups associated with different levels of RN infestation clustered distinctly from fungal groups (**Figures 3A, B**). In the beta diversity plot, samples with similar bacterial composition profiles were clustered together, while those with differing profiles were positioned further apart, effectively illustrating the overall bacterial composition. The microbial diversity within the fungal Group D clustered and overlapped with groups A and C across various RN infestation levels (**Figure 3B**). The presence of RN infestation notably influenced the clustering patterns of the samples and their microbial classification (Nielsen et al., 2023).

### 3.4 Relative abundance

#### 3.4.1 Phylum level

**Figure 4:**
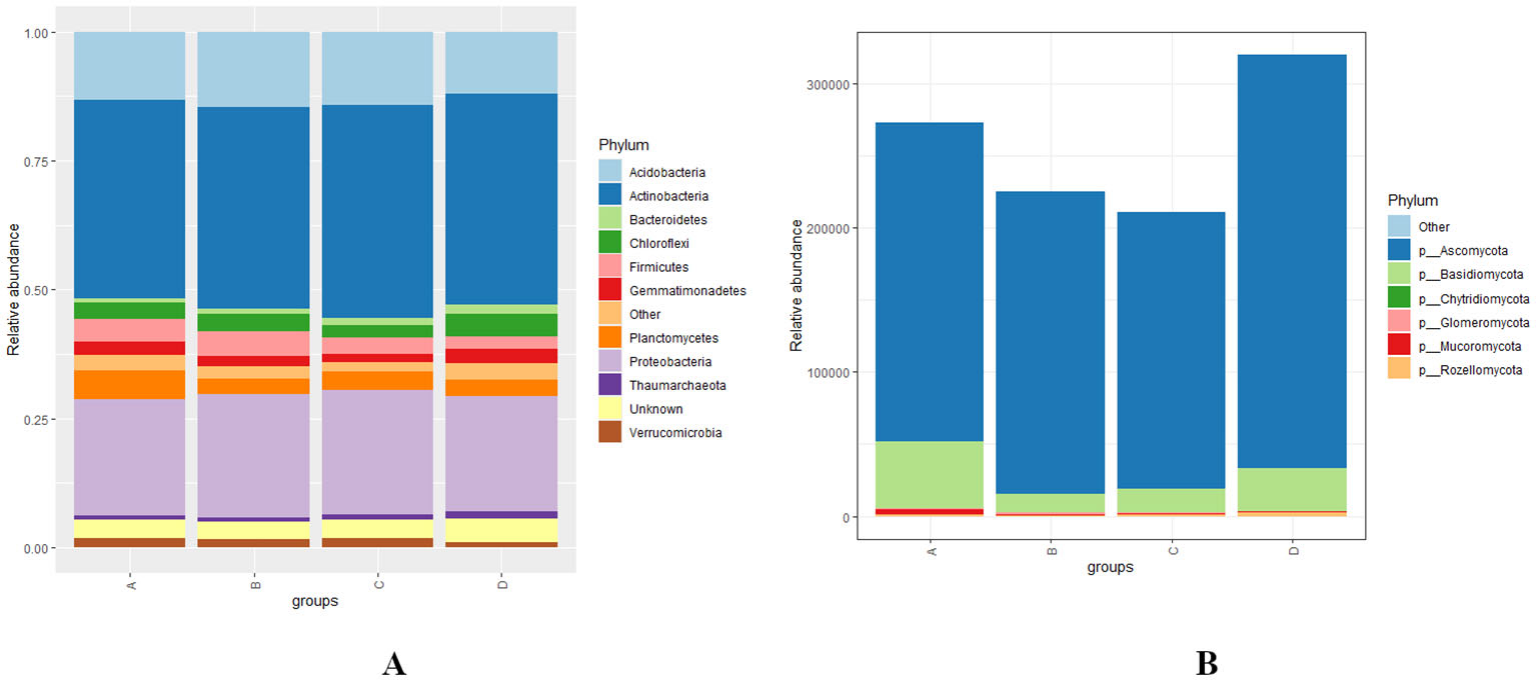
**(A, B)** Distribution and relative abundance of bacterial and fungal phyla at different levels of RN Infestation with statistical significance (*p* < 0.01). Group A-RN Not-Detected, Group B- RN Low Infestation, Group C- RN Medium Infestation, Group D- RN High Infestation.

At the phylum level, the bacterial phyla *Actinobacteria, Proteobacteria, Acidobacteria*, and *Planctomycetes* exhibited high relative abundances, followed by *Chloroflexi, Firmicutes, Gemmatimonadetes, Verrucomicrobia*, and *Bacteroidetes* across various levels of RN infestation with a statistical significant difference (*p* < 0.01) (**Figure 4A**). In all four groups A, B, C, and D-*Actinobacteria, Proteobacteria*, and *Acidobacteria* demonstrated similar patterns of relative abundance. Notably, in Group A, *Planctomycetes* were more abundant than in the other groups, while *Firmicutes* showed higher relative abundance in Group B. In Group C, *Verrucomicrobia* was the most abundant, whereas the highest abundances of *Chloroflexi* and *Bacteroidetes* observed in Group D (**Figure 4A**). The fungal community composition indicates that *Ascomycota* is the most predominant phylum, followed by *Basidiomycota, Mucoromycota*, and *Rozellomycota* with a significant statistical difference (*p* < 0.01). Specifically, *Ascomycota* was the dominant phyla in Group D, while *Basidiomycota* and *Mucoromycota* were most abundant in Group A. However, *Mucoromycota* was the least abundant phyla across all groups except for Group A, highlighting distinct compositional differences among the groups (**Figure 4B**).

#### 3.4.2 Family level

**Figure 5:**
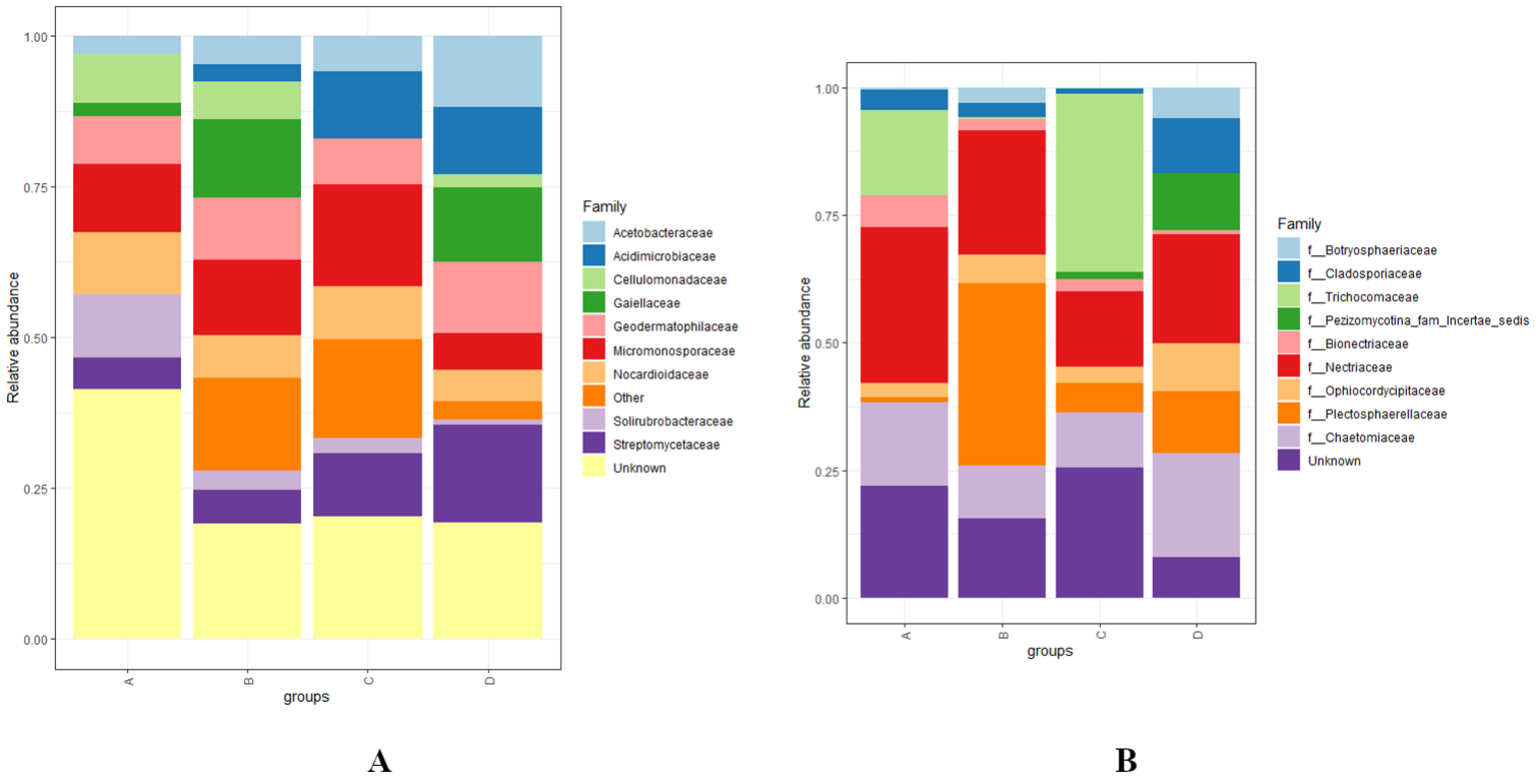
**(A, B)** Distribution and relative abundance of bacterial and fungal communities at the family level across various levels of RN infestation with statistical significance (*p* < 0.01). Group A-RN Not-Detected, Group B- RN Low Infestation, Group C- RN Medium Infestation, Group D- RN High Infestation.

At the family level, the bacterial families *Solirubrobacteraceae, Nocardiodaceae, Micromonosporaceae, Acidimicrobiaceae, Acetobacteraceae, Streptomycetaceae*, and *Geodermatophilaceae* were the most abundant with the statistical significance of *p* < 0.01 across various levels of RN infestation. In Group A, *Solirubrobacteraceae, Cellulomonadaceae*, and *Nocardiodaceae* were observed as the most abundant bacterial families, while *Gaiellaceae* was the least identified, and *Acidimicrobiaceae* was completely absent. In Group B, *Gaiellaceae* emerged as the most abundant family, whereas *Acidimicrobiaceae* was the least abundant. In Group C, *Micromonosporaceae* and *Acidimicrobiaceae* were the most abundant, while *Cellulomonadaceae* and *Gaiellaceae* were the least abundant families. In Group D, *Acetobacteraceae, Streptomycetaceae*, and *Geodermatophilaceae* were the most abundant bacterial families. Remarkably, *Solirubrobacteraceae* was also found to be the least abundant in both groups C and D (**Figure 5A**). The diversity of fungal families was highlighted by the predominance of *Nectriaceae, Bionectriaceae, Plectosphaerellaceae, Cladosporiaceae, Chaetomiaceae*, and *Ophiocordycipillaceae* across various levels of RN infestation with the statistical significance of *p* < 0.01. In Group A, *Nectriaceae* and *Bionectriaceae* were identified as the most abundant families, *Plectosphaerellaceae* was the least represented, and *Botryosphaeriaceae* and *Pezizomycotina-farm-incertae sedis* were not detected. In Group B, *Plectosphaerellaceae* was the most abundant family and *Trichocomaceae* and *Pezizomycotina-farm-incertae sedis* were not observed. While in group C, *Trichocomaceae* was the most prevalent and *Cladosporiaceae* was the least abundant. Notably, *Bionectriaceae* was the least abundant in both groups B and C. In Group D, *Cladosporiaceae*, *Pezizomycotina-farm-incertae sedis*, *Chaetomiaceae, Botryosphaeriaceae, Ophiocordycipillaceae* were the most abundant and *Bionectriaceae* was the least abundant fungal families identified. Similarly to Group B, *Trichocomaceae* was also not observed in Group D (**Figure 5B**).

#### 3.4.3 Genus level

**Figure 6:**
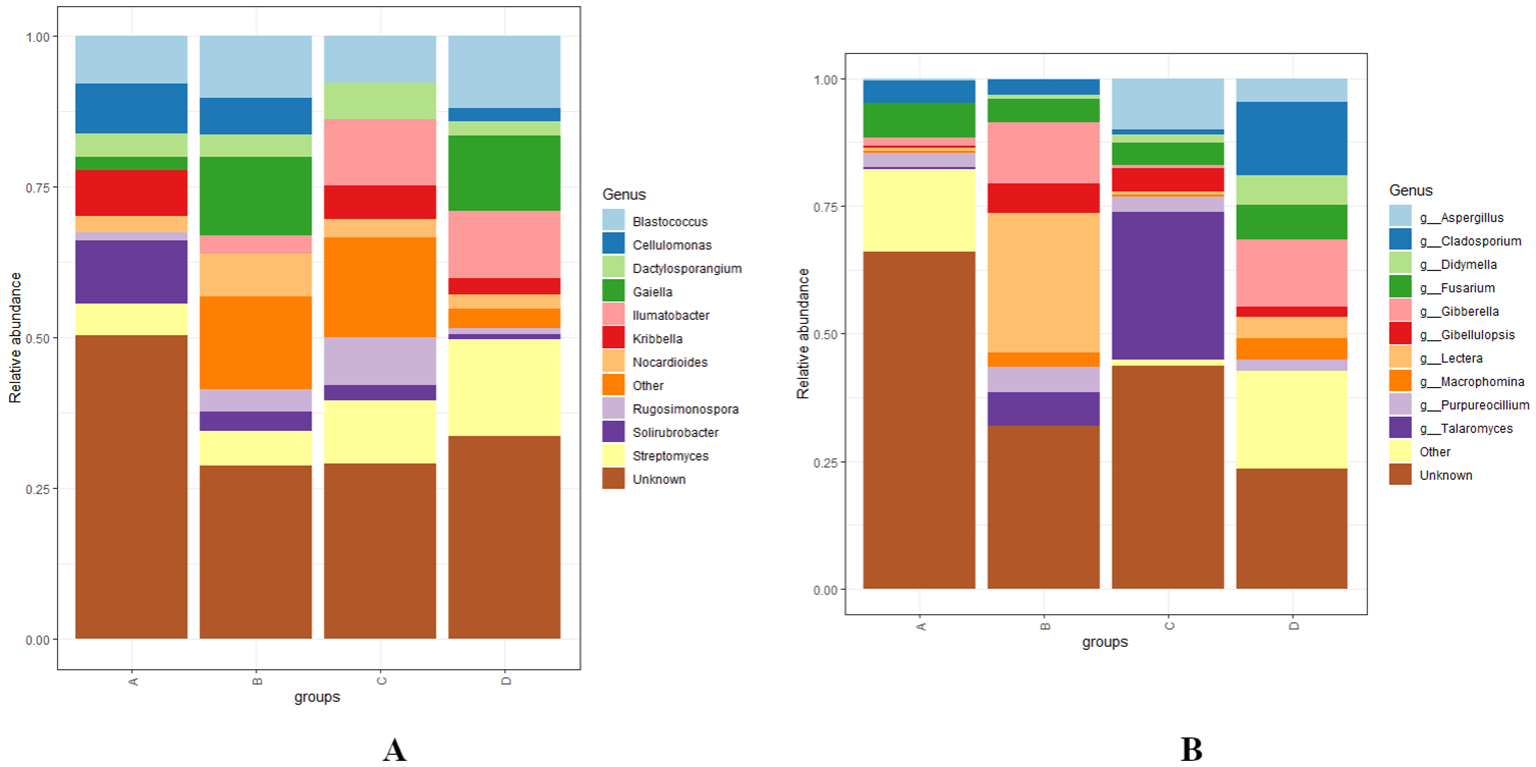
**(A, B)** Distribution and relative abundance of bacterial and fungal genera at different levels of RN Infestation with statistical significance (*p* < 0.01). Group A-RN Not-Detected, Group B- RN Low Infestation, Group C- RN Medium Infestation, Group D- RN High Infestation.

At the genus level, *Solirubacter, Nocardioides, Dactylosporangium, Rugosimonospora, Blastococcus, Streptomyces*, and several unclassified genera were identified as the most abundant bacterial genera across different levels of RN infestation with statistical significance, *p* < 0.01. In Group A, *Kribbella, Cellulomonas*, and *Solirubrobacter* were identified as the most abundant, *Rugosimonospora* was found to be the least abundant and *Illumatobacter* was not detected. In Group B, *Gaiella* and *Nocardioides* were identified as the most abundant and *Illumatobacter* was identified as the least abundant genera. In contrast to Group A, *Kribbella* was absent in Group B. In Group C, *Ilumatobacter, Dactylosporangium*, and *Rugosimonospora* were identified as the most abundant while *Gaiella* and *Cellulomonas* were completely absent. In Group D, *Blastococcus* and *Streptomyces* were detected as most abundant. Interestingly, *Solirubacter* was observed as the least abundant genera in groups C and D (**Figure 6A**). The diversity of fungal genera composition was statistically significant at *p* < 0.01 with predominant genera including *Fusarium, Lectera, Gibellulopsis, Purpureocillum, Cladosporium, Fusarium, Macrophomina* and several unclassified genera across various levels of RN infestation. In Group A, *Fusarium* was identified as the most abundant and *Talaromyces* was the least represented genera. In Group B, *Lectera, Gibellulopsis*, and *Purpureocillum* were identified as the most abundant and *Aspergillus* was not detected. In addition, *Didymela* was not detected in both Group A and B. In Group C, *Talaromyces* and *Aspergillus* were identified as the predominant genera and *Lectera* was identified as the least abundant genera. In Group D, *Cladosporium, Didymella, Fusarium*, and *Macrophomina* were abundant, *Gibellulopsis* was the least abundant and *Talaromyces* was not detected (**Figure 6B**).

### 3.5 Venn diagram

**Figure 7:**
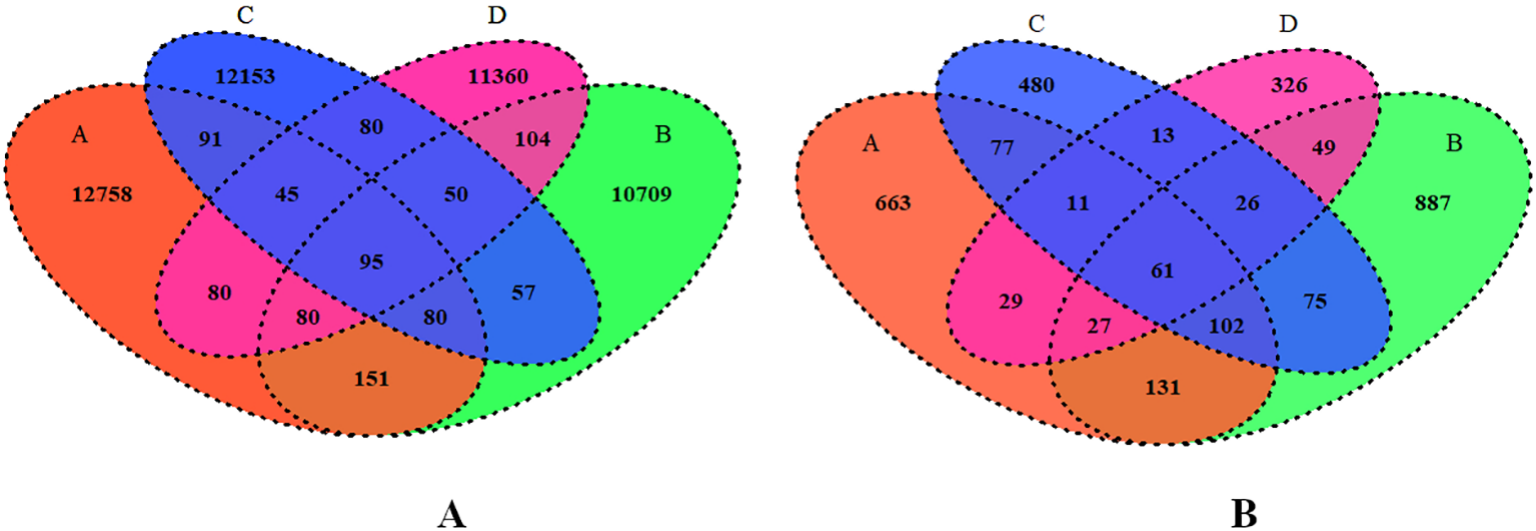
**(A, B)** Venn diagram showing total numbers of shared bacterial and fungal ASVs across various levels of RN Infestation. Group A-RN Not-Detected, Group B- RN Low Infestation, Group C- RN Medium Infestation, Group D- RN High Infestation.

To further understand the bacterial and fungal distribution within the microbiota, shared and unique ASVs across different groups under comparison were analyzed using a Venn diagram (**Figures 7A, B**). In total, 47,893 bacterial and 3,409 fungal ASVs were identified among all groups, with 95 ASVs shared among all bacterial groups and 61 ASVs shared among all fungal groups. For the bacterial ASV’s, 12,758, 10,709, 12,153 and 11,360 unique ASVs were identified in Groups A, B, C and D, respectively (**Figure 7A**). For the fungal ASVs, 663, 887, 480, and 326 unique ASVs were identified in Groups A, B, C and D, respectively. (**Figure 7B**).

### 3.6 Differential relative abundance analysis

**Figure 8:**
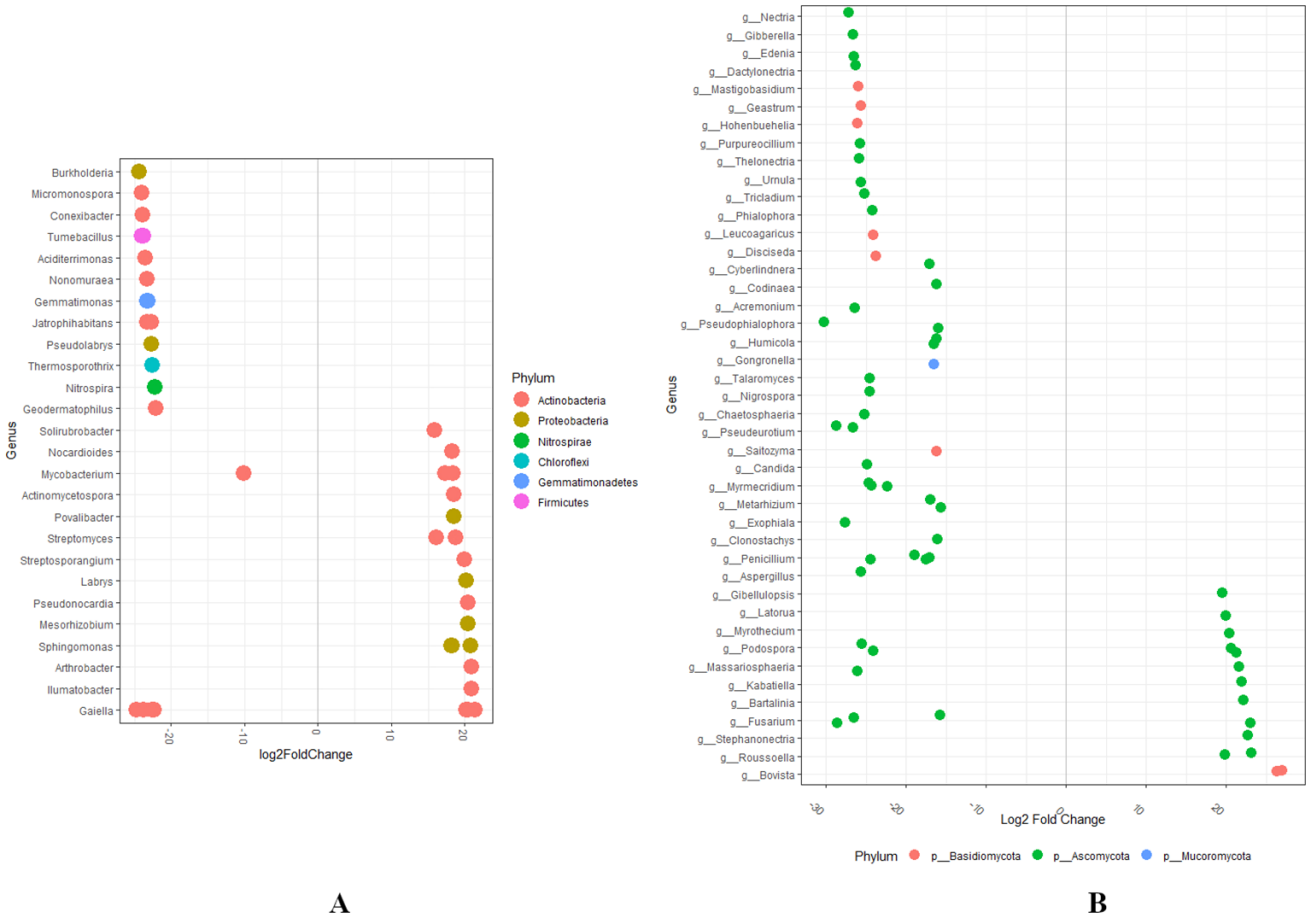
**(A, B)** Differential abundance analysis of bacterial and fungal microbial genera at the phylum level was conducted by comparing Group A (RN Not-Detected) with Group D (RN High Infestation) using the DESeq2 (statistical significance, *p* < 0.05).

To investigate variations in the relative abundance of bacterial and fungal genera, we analyzed the dataset using log2 fold change by comparing Group A to Group D. This differential abundance analysis revealed significant changes in the bacterial and fungal microbial communities (**Figure 8A**). Several bacterial genera exhibited a marked increase (*p* < 0.05; log_2_FC > 2) in the abundance of *Streptosporangium, Labrys, Pseudonocardia, Mesorhizobium, Sphingomonas, Arthrobacter, Ilumatbacter*, and *Gaiella* in Group D, compared to Group A. Contrastingly, a significant decrease in the abundance of bacterial genera such as *Burkholderia, Micromonospora, Jatrophihabitans, Gaiella*, and *Mycobacterium* was observed (**Figure 8A**). The fungal genera exhibited a marked increase in the abundance of *Gibellulopsis*, *Latorua*, *Myrothecium*, *Podospora*, *Russoella*, and *Bovista* in Group D relative to Group A. Contrastingly, a significant decrease in the abundance of *Tricladium*, *Phialophora*, *Humicola*, *Talaromyces*, *Nigrospora*, *Chaetosphaeris*, *Candida*, *Myrmecredium*, *Penicillium*, and *Aspergillus* were observed (**Figure 8B**).

### 3.7 Core microbiome

**Figure 9:**
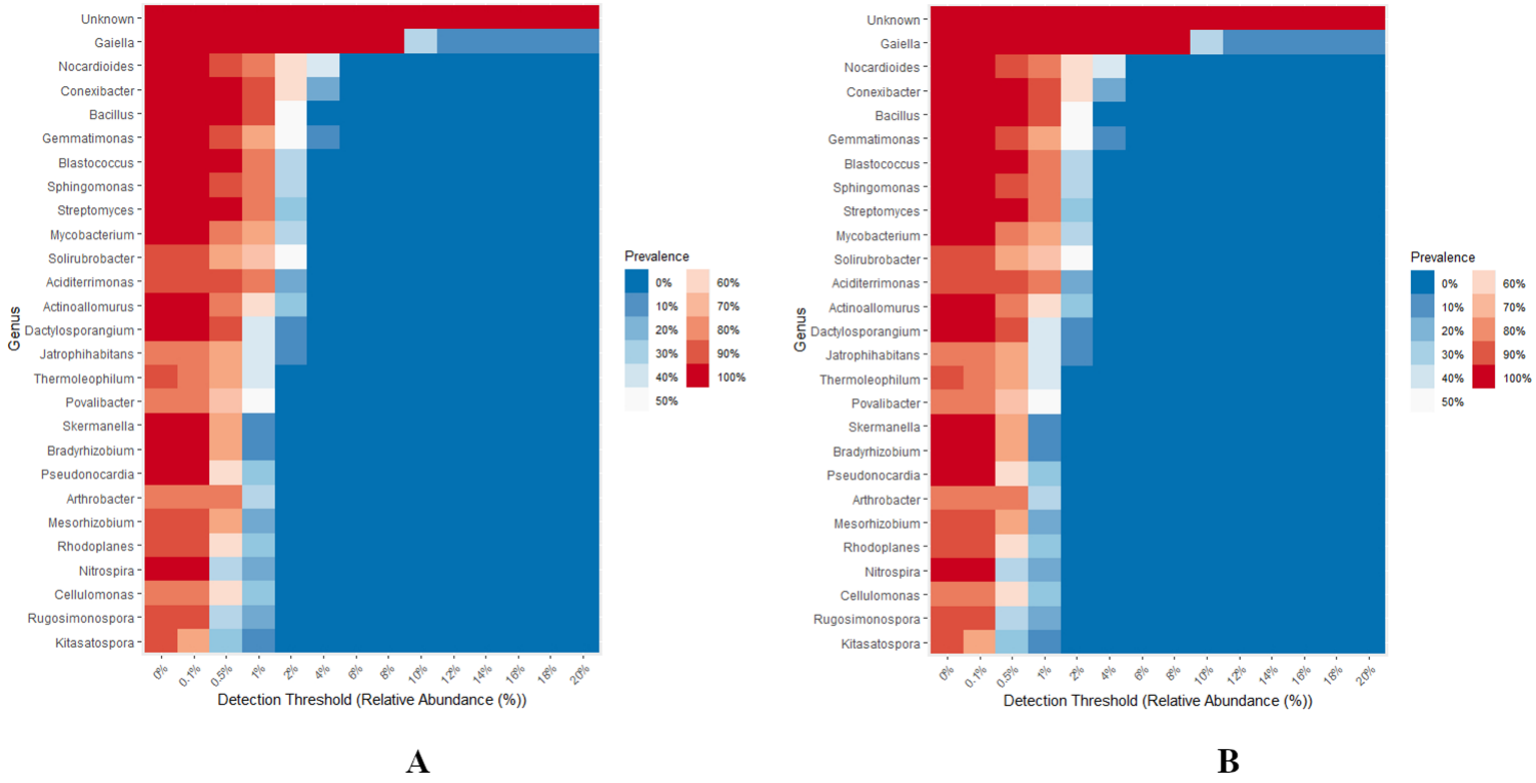
**(A, B)** Heat map showing core bacterial and fungal genera across different levels of RN Infestation. The plot compares the prevalence of genus in samples across varying levels of abundance. Only the genera with minimum prevalence of 0.2 at 0.001 abundance are plotted.

A total of 27 bacterial and 38 fungal genera were identified as part of the core microbiome, across all groups, considering a minimum of 0.1% abundance observed among > 20% of the samples (**Figures 9A, B**). Notably, *Gaiella* emerged as the core bacterial genera, exhibiting a prevalence of 100% and a relative abundance of 8%. Additionally, *Conexibacter*, *Bacillus*, *Blastococcus*, and *Streptomyces* were recognized as core bacterial genera, each showing a relative abundance of 0.5% across all groups. Furthermore, *Sphingomonas*, *Mycobacterium*, *Actinoallomurus*, *Dactylosporangium*, *Skermanella*, *Bradyrhizobium*, *Pseudonocardia*, and *Nitrospora* were classified as core bacterial genera, each with a relative abundance of 0.1% across all groups. The remaining bacterial genera displayed an abundance of 0.1% with a prevalence ranging from 70-90% (**Figure 9A**). In terms of fungal genera, *Fusarium* was recognized as a core member of the microbiome, with a prevalence of 100% and a relative abundance of 1%. *Aspergillus* was also identified as a core fungal genus, with a relative abundance of 0.5% across all groups. Moreover, *Gibberella*, *Cladosporium*, and *Lactera* were categorized as core fungal genera, each exhibiting a relative abundance of 0.1% across all groups. The remaining fungal genera had an abundance of 0.1% with a prevalence range of 30-90% (**Figure 9B**).

### 3.8 Network plots of bacterial and fungal phyla

**Figure 10:**
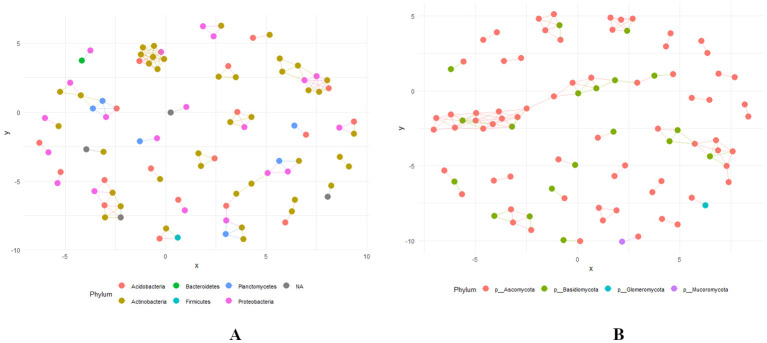
**(A, B)** Network Plot showing the relationship based on Jaccard Distance at phylum level with bacterial and fungal communities across various levels of RN Infestation (Significant (*p* < 0.01) correlation). Group A-RN Not-Detected, Group B- RN Low Infestation, Group C- RN Medium Infestation, Group D- RN High Infestation.

Network plots serve as a powerful visual tool for understanding the relationships among various bacterial and fungal phyla with statistical correlation, *p* < 0.01. In these plots, nodes represent different bacterial phyla, such as *Actinobacteria, Acidobacteria, Bacteroidetes, Firmicutes, Planctomycetes*, and *Proteobacteria* and. fungal phyla such as *Ascomycota, Basidiomycota, Glomeromycota*, and *Mucoromycota*. Edges represent the degree of similarity in taxonomic composition based on shared ASVs (**Figures 10A, B**). A tight clustering of bacterial nodes was identified among *Actinobacteria, Acidobacteria*, and *Proteobacteria* indicating a high degree of similarity and co-occurrence. This clustering suggests niche sharing, potential ecological interactions and functional roles in the ecosystem (**Figure 10A**). Conversely, isolated bacterial nodes were found in *Bacteroidetes, Firmicutes*, and *Planctomycetes* with moderate connections indicating distinct ecological dynamics or a specialized function within the community. The nodes of fungal phyla like *Ascomycota* and *Basidiomycota* were closely connected, implying similar ecological roles within the microbial community. Contrastingly, nodes of the *Glomeromycota* and *Mucoromycota* appear to be more isolated, indicating varied functional roles and ecological dynamics (**Figure 10B**).

## 4 Discussion

This study advances our understanding of the rhizosphere-associated microbiome to RN infestations by revealing bacterial and fungal richness and evenness shifts across varied infestation levels. Our analysis shows that nematode infestation significantly affects the composition and diversity of rhizospheric microbiota, with notable shifts across infestation levels. Specifically, Group A has the highest abundance of bacterial phyla *Planctomycetes* and fungal phyla *Basidiomycota*, *Mucoromycota*, and *Ascomycota*. Group D was characterized by the predominance of bacterial phyla *Chloroflexi* and fungal phyla *Ascomycota*. These shifts highlight the resilience and adaptability of the microbiome in response to RN, suggesting a complex interplay between microbial community dynamics and nematode presence. The observed alterations in microbial composition may enhance plant defense mechanisms, as specific microbial taxa can facilitate nutrient acquisition and promote plant growth during stress conditions (Zhang et al., 2020).

The analysis of alpha diversity metrics revealed distinct patterns in microbial community composition corresponding to varying reniform nematode (RN) infestation levels. Notable differences were observed in both bacterial and fungal richness and evenness. Group D showed higher bacterial richness and evenness, while Group A had lower bacterial richness. These results are consistent with those of Lundberg et al. (2020b), that reported increased pest incidence like nematode infestations, can lead to more diverse microbial communities due to improved nutrient availability during stress. Conversely, Group A’s fungal communities showed higher richness and evenness, indicating a more stable microbial community associated with healthy soil ecosystems, as reported by Yuan et al. (2020b). Moreover, Group B revealed elevated fungal richness in observed species, suggesting that even moderate nematode infestations can modify root exudation patterns that may subsequently benefit plant health, as Topalovic et al. (2020) indicated. Conversely, in Group D, both fungal richness and evenness were diminished, consistent with previous studies indicating that increased nematode infestation can disrupt the microbial balance, favoring opportunistic species and resulting in a decline in overall microbial diversity, as reported by Zhang et al. (2019).

The Shannon rarefaction curves generated in our study indicate that microbial diversity responds distinctly to varying levels of RN infestation for both bacterial and fungal communities. The observation that the Shannon index plateaued after reaching 30,000 sequences supports the notion that our sequencing efforts sufficiently captured the microbial diversity present in the samples, corroborating similar studies suggested by Olesen and Simmelsgaard (2019) and Chao et al. (2014), which suggest that adequate sampling depth is crucial for reliable diversity assessments. Group D exhibited higher microbial diversity, which aligned with previous reports showed that microbial diversity during nematode infestations (Huang et al., 2022; Zhang et al., 2023). In Group D, the values of the Shannon index showed a higher microbial diversity within bacterial communities suggesting a robust and resilient bacteria that is capable of sustaining functions that are critical for nutrient cycling and plant growth (Wang et al., 2023). Fungal communities showed distinct patterns, with Group A having the highest diversity, suggesting low RN infestation levels may favor a diverse fungal community, potentially enhancing plant health and nutrient uptake (Martínez-García et al., 2023). In contrast, lower fungal diversity indicates that biotic stress negatively impacts the dynamics of fungal communities (Liu et al., 2023).

The PERMANOVA analysis of weighted UniFrac distances (*p* < 0.01) showed differences in the microbial composition across all groups, suggesting that nematode-induced biotic stress is the primary factor driving microbial community shifts. As spatial variation could potentially influence microbial communities, our experimental design ensured that all samples were collected from similar environmental conditions, minimizing spatial variability. In addition, nematode behavior can also cause significant shifts in microbial communities, independent of spatial variation in the sampling environment (Zhang et al., 2019). In the principal coordinate analysis (PCoA), bacterial groups associated with different levels of RN infestation clustered distinctly from fungal groups (**Figures 3A, B**), a similar pattern reported by Raaijmakers et al., 2009; Zhao et al., 2018; Zhang et al., 2019. Interestingly, an overlap of fungal communities was observed among Groups A, C, and D, suggesting the functional stability of specific fungal taxa despite the fluctuations in biotic stress (Nielsen et al., 2023).

At the phylum level, microbial community composition reveals significant insights into the dynamics of bacterial and fungal populations in response to varying levels of RN infestation. Our results indicate that the bacterial phyla *Actinobacteria, Proteobacteria*, and *Acidobacteria* were highly abundant across all groups, consistent with earlier studies that highlighted their roles in nutrient cycling and plant growth promotion in soil ecosystems (Fierer et al., 2007). Specifically, *Actinobacteria* are known for their capacity to degrade organic matter and contribute to soil health (Jansson and Hofmockel, 2009). Notably, *Planctomycetes* were identified as abundant phyla in Group A, which may indicate their role in nitrogen cycling (Rao and Rao, 2016). Similarly, the higher relative abundance of *Firmicutes* in Group B, *Verrucomicrobia* in Group C, and *Chloroflexi* and *Bacteroidetes* in Group D suggests that these bacteria may play a crucial role in maintaining soil health and nutrient cycling and also associated with the breakdown of complex organic compounds thus enriching the soil (Guan et al., 2018; Ding et al., 2024; Wang et al., 2019). The higher abundance of *Ascomycota* in Group D is important for improving soil health (Lücking et al., 2017). In Group A, the higher abundance of *Basidiomycota* and *Mucoromycota* reflects a potential competitive advantage of these phyla (Zong et al., 2021). A lower abundance of *Mucoromycota* was observed among all groups except Group A, suggesting that the resilience of fungal groups varies with nematode infestation levels (Nielsen et al., 2023).

In Group A, the dominance of *Solirubrobacteraceae* and *Cellulomonadaceae* families was observed, similar to what was reported by Youssef et al., 2015, suggesting their role in cellulose degradation. *Gaiellaceae* was the most abundant family observed in Group B, indicating its adaptability to varied environmental conditions (Vasquez et al., 2019). In contrast, *Acidimicrobiaceae* was the least abundant family in both Group A and B, which may indicate competitive exclusion by more dominant families in less disturbed soils (Sang et al., 2019). In Group C, *Micromonosporaceae* and *Acidimicrobiaceae* were the most abundant families, which can play a crucial role in secondary metabolite production and mitigate stress impacts (Liu et al., 2020). Group D exhibited increased abundance with *Acetobacteraceae*, *Streptomycetaceae*, and *Geodermatophilaceae* families, which are generally associated with nutrient cycling (Meyer et al., 2022). The predominance of *Nectriaceae* and *Bionectriaceae* in Group A suggests their vital role in plant health, nutrient mobilization, and soil ecology (Kurtzman et al., 2018). In Group B, the *Plectosphaerellaceae* family was the most abundant, and *Trichocomaceae* was the least abundant, suggesting a potential vulnerability of specific fungal taxa even with mild RN infestation (Zhao et al., 2021). In Group D, the increased abundance of *Cladosporiaceae* and *Chaetomiaceae* suggests the stability of these fungal communities that may affect plant-microbe interactions and overall soil health (Nielsen et al., 2023).

In Group A, the prevalence of *Kribbella*, *Cellulomonas*, and *Solirubacter* and the absence of *Illumatobacter* indicates that specific genera were dominant and are potentially involved in cellulose degradation and organic matter breakdown (Fierer et al., 2007; Liu et al., 2020). In Group B, the relative abundance of *Gaiella* and *Nocardioides* reflects a shift in community dynamics, which can enhance nutrient availability and promote plant growth (Zhang et al., 2018). In Group C, a higher abundance was observed in *Illumatobacter*, *Dactylosporangium*, and *Rugosimonospora*, indicating their resilience and adaptability in response to biotic stress (Pester et al., 2010). A decrease in the abundance of *Gaiella* and *Cellulomonas* suggests that certain nematodes can affect root-associated microbial communities essential for maintaining plant health (Jousset et al., 2017). In Groups C and D, *Solirubacter* was the least abundant genus potentially sensitive to higher RN infestation levels (Zhang et al., 2020). In Group A, *Fusarium* is the most abundant genus that plays a dual role as a pathogen and beneficial organism (Gams et al., 2011). Lower abundance of *Didymella* in Groups A and B may indicate shifts in community structure linked to nematode infestation levels (Grondahl et al., 2021). In Group C, the dominance of *Talaromyces* and *Aspergillus* suggests that specific fungal taxa might thrive in moderate RN infestation, likely due to their saprophytic capabilities and ability to decompose organic matter (Jaklitsch et al., 2016). The abundance of *Cladosporium*, *Didymella*, *Fusarium*, and *Macrophomina* in Group D may support the complex interactions in soil systems. A lower abundance of *Talaromyces* reflects competitive exclusion caused by higher nematode loads (Zhang et al., 2022).

Among the bacterial communities, 95 shared ASVs among all groups indicate a core set of taxa that persists across varying environmental conditions (Shade et al., 2012a). Identifying 61 shared fungal ASVs underscores the potential for specific fungal taxa to adapt and thrive in diverse soil environments (Glassman et al., 2017). The high number of unique ASVs in Group A (12,758) may facilitate niches that support a wider range of bacterial diversity that may be involved in improving nutrient availability and fostering ecological interactions (Lauber et al., 2009). A lower number of unique ASVs (10,709) were observed in Group B compared to Group A, reflecting a substantial diversity that may stimulate competitive interactions under mild RN infestation (Santos et al., 2018). In Group C, 12,153 unique ASVs identified belonged to diverse bacterial communities with specific functional roles in soil and plant health (Friedman and Alm, 2012). In Group D, relatively lower bacterial communities (11,360 unique ASVs) identified were possibly due to the microbial and pest competition for the available nutrients (Wagg et al., 2014). A higher diversity of fungal ASVs was identified in Group A (663) compared to Group D (326), suggesting that specific fungal communities may be more resilient to biotic stress, highlighting the interplay between fungal diversity and plant health (Zhao et al., 2021).

By suppressing plant defenses, nematodes may inadvertently alter the composition of microbial communities in the rhizosphere. This can lead to an increase in stress-resistant bacterial taxa better adapted to the modified environment. Moreover, the weakened plant defense system can also facilitate the colonization and proliferation of secondary pathogens, including bacteria, which may further exploit the compromised plant defenses (Shade et al., 2012b; McGuire et al., 2017). The increased abundance of genera such as *Streptosporangium, Labrys, Pseudonocardia, Mesorhizobium, Sphingomonas*, and *Arthrobacter* in Group D indicates a shift in stress-resistant taxa. Various ecological interactions and environmental factors influence the shift in specific microbial populations such as *Burkholderia, Micromonospora, Jatrophihabitans, Gaiella*, and *Mycobacterium* to selective pressures from nematode infestations. The change in such bacterial genera in Group D may be attributed to the selective pressure exerted by higher levels of RN infestation. *Burkholderia* species, particularly *B. seminalis*, have shown potential as biocontrol agents against nematodes like *Meloidogyne enterolobii*. Studies have demonstrated that specific concentrations of *B. seminalis* can exhibit ovicidal activity, reducing nematode egg viability and thus controlling nematode populations (Moreira et al., 2024). *Burkholderia* is highly attractive to certain nematodes, such as *M. incognita*, which can increase nematode aggregation around these bacteria. This attraction can influence the dynamics of nematode populations and their interactions with other microbial communities (Tahseen and Clark, 2014). Fungal genera such as *Gibellulopsis, Latorua, Myrothecium, Podospora, Russoella*, and *Bovista* employ a variety of mechanisms to suppress nematode infestations. These fungi are part of a broader group known as nematophagous fungi, which are recognized for their ability to control nematode populations through diverse strategies such as mechanical trapping, endoparasitism, systemic resistance, enzymatic degradation, and toxic metabolite production (Noweer, 2020). Interestingly, a significant increase in the abundance of these fungal genera was observed in Group D. A significant decrease in the fungal genera such as *Tricladium, Phialophora, Nigrospora*, and *Candida* was observed. The presence of some nematophagous fungi can potentially reduce the prevalence of non-nematophagous genera like *Tricladium* and *Phialophora* (Mo et al., 2023).

The presence of nematodes in the soil leads to increased alkaline phosphomonoesterase (ALP) activity, directly linked to higher phosphorus availability. This enhanced nutrient cycling provides a competitive edge to bacteria like *Gaiella*, that can efficiently utilize the available phosphorus (Zheng et al., 2022). The core bacterial genus *Gaiella* emerged as a dominant genus, exhibiting a prevalence of 100% and a relative abundance of 8%, indicating the crucial role in phosphorus recycling. The specific mechanism by which *Conexibacter, Bacillus, Blastococcus*, and *Streptomyces* bacteria outcompete other microbial communities under nematode infestation involves a combination of biochemical and ecological strategies. The presence of these bacteria with a relative abundance of 0.5% suggests their possible role in survival and proliferation in the rhizosphere by competing against the nematodes. Nematode-induced nutrient cycling significantly impacts the selective advantage of bacteria such as *Sphingomonas, Mycobacterium*, and *Actinoallomurus* in mixed microbial communities, as identified in Group D, with a relative abundance of 0.1%. Through predation and feeding activities, nematodes influence the availability of nutrients like nitrogen and phosphorus, affecting bacterial community dynamics and competitive interactions. This process can enhance the growth and activity of specific bacterial taxa, providing them with a competitive edge in nutrient-limited environments (Zheng et al., 2022). In Group D, *Fusarium* was observed with a 100% prevalence and a relative abundance of 1%. This dominance may be due to the complex relationship between *Fusarium* and nematodes, including antagonistic and synergistic interactions. These interactions can vary based on environmental conditions and the specific species involved (Siddiqui and Aziz, 2024). In Group D, *Aspergillus*, *Gibberella, Cladosporium*, and *Lactera* were identified with a relative abundance of 0.1%. These fungi can play various roles, from being parasitic to nematodes to acting as part of a broader soil microbiome that influences nematode behavior and survival.

The presence of nematodes alters the soil environment, affecting the bacterial community structure and promoting the clustering of specific bacterial taxa that can thrive under these conditions. We observed tight clustering among bacterial nodes, particularly in *Actinobacteria, Acidobacteria*, and *Proteobacteria* in the core microbiome. This clustering is likely influenced by the competitive exclusion of less adapted bacterial clades and the selective pressures exerted by the nematodes and the altered soil environment (Yergaliyev et al., 2020). The isolation of nodes within *Bacteroidetes, Firmicutes*, and *Planctomycetes* under plant parasitic nematode infestation is driven by complex interactions between the nematodes, the plant host, and the microbial communities in the rhizosphere. These interactions are influenced by the nematode’s life cycle, the plant’s response to infestation, and the environmental conditions in the soil (Yergaliyev et al., 2020). Forming closer tier networks within *Ascomycota* and *Basidiomycota* in response to RN levels provides significant evolutionary advantages. These fungi have evolved mechanisms that enhance their survival and ecological roles by forming intricate networks optimized for resource acquisition, defense, and symbiosis with host plants. Such networks are essential in environments with higher nematode levels, as they help mitigate the damage caused by these pests (Kitagami and Matsuda, 2024). Conversely, the formation of isolated networks with *Glomeromycota* phylum, particularly arbuscular mycorrhizal fungi (AMF), such as species from the genus *Glomus*, under RN infestation can be attributed primarily to their potential role in enhancing plant resistance and growth. These fungi possibly establish symbiotic relationships with plant roots, thereby improving nutrient uptake and serve as a biological control against nematodes (Chaerani and Ginting, 2023).

### 4.1 Potential pitfalls associated with soil microbial profiling studies

Conducting soil microbial analysis presents several potential pitfalls researchers must navigate to ensure accurate and reliable results. One of the significant challenges in such studies is the inherent heterogeneity of soil, which complicates the sampling process. Soil is a dynamic entity with varying microbial populations, and sampling must be statistically sound to capture this diversity accurately. Additionally, the physicochemical properties of soil, such as pH, and organic content can significantly influence microbial community composition and activity, necessitating careful consideration and control in experimental designs. The rapid changes in microbial populations during sample handling and storage also necessitate prompt transfer to laboratories in order to prevent alterations in microbial activity. Also, there is difficulty in estimating the concentration and activity of mixed microbial populations due to their heterogeneous nature and varying metabolic rates. Traditional methods like fluorescence and spectrophotometry have limitations, and microscopy is often recommended for more accurate measurements. Moreover, integrating molecular techniques in soil microbial analysis while offering advanced insights requires careful interpretation to avoid misrepresenting microbial diversity and function. Furthermore, the lack of soil-specific reference databases for metagenomic classifiers poses a challenge in accurately profiling soil microbiomes. Custom databases, optimized classifiers with improved accuracy in taxonomic classification, and tailored bioinformatic pipelines are required. Lastly, sharing data and establishing standard guidelines are crucial for reproducibility and meta-analyses, which can enhance the understanding of soil microbial communities and their ecological roles. The experimental design of this study was structured to address the potential pitfalls by adhering to the Alabama Cooperative Extension System’s protocols.

## 5 Conclusion

The study explores the relationships between reniform nematode (RN) infestation and the rhizosphere microbiome dynamics in cotton soils. It finds that RN infestation affects the diversity and composition of microbial communities, which in turn enhances plant resistance to biotic stress. These microbial shifts also impact vital biogeochemical cycles important for soil fertility. Furthermore, the research delineates specific bacterial and fungal taxa associated with RN infestation, indicating potential approaches for biological control and soil management. Our findings underscore the importance of comprehending plant-microbe-nematode interactions to formulate integrated pest management strategies that promote sustainable cotton production.

## 6 Future directions

Designing individual and integrated experiments to understand tripartite interactions among plant-nematode-soil microbiomes is critical during Reniform nematode infestation. Growing and maintaining specific bacterial or fungal pure cultures identified during RN infestation will improve our understanding of these unique microbial species’ functional roles. This knowledge will facilitate the exploration of associated plant defense mechanisms, potentially leading to the development of targeted biological control strategies. Also, investigating the interactions between nematodes, rhizosphere microbiomes, and different cotton genotypes using multi-omic approaches could enhance our understanding of metabolite degradation, nutrient availability in soil, host-parasite competition, and selective pressures exerted on microbial populations during nematode infection. To further strengthen our knowledge, pot culture studies under controlled conditions with different genotypes play a crucial role in examining microbial shifts during RN infection to comprehend the link between microbial dynamics and plant resistance. Longitudinal studies assessing the impact of various nematode management practices on microbial community composition and soil health are essential. Applying these approaches to other plant-nematode systems will support our findings. This will help us understand broader ecological effects and promote sustainable farming practices.

## Data Availability

The datasets generated for this study can be found in the NCBI Sequence Reads Archive (SRA) with the accession numbers SAMN45928254 - SAMN45928265 for 16S rRNA and SAMN45929394 - SAMN45929405 for ITS, under the BioProject, PRJNA1201180.

## Supplementary material

Supplementary Figure 1 North Alabama map showing levels of Reniform Nematode (RN) Infestation in four selected counties.

## References

[B1] Abarenkov K. Tedersoo L. Nilsson R. H. Veldre V. Paap T. Zirk A.. (2023). UNITE QIIME release for fungal ITS sequencing, with improved taxonomic assignment, analysis, and visualization. Fungal Diversity 114, 319–324. doi: 10.1007/s13225-023-00448-z

[B2] Alabama Cooperative Extension System (2020). Soil Survey and Soil Types of North Alabama (Auburn, AL: Alabama A&M and Auburn University).

[B3] Allen E. E. Banfield J. F. (2021). Community genomics in microbial ecology. Nat. Rev. Microbiol. 19, 233–244. doi: 10.1038/s41579-020-00456-1 15931167

[B4] Andersen K. S. Kirkegaard R. H. Karst S. M. Albertsen M. (2018). ampvis2: An R package to analyse and visualize 16S rRNA amplicon data. BioRxiv, 299537. doi: 10.1101/299537

[B5] Auguie B. (2017). gridExtra: Miscellaneous functions for “grid” graphics. R package version 2.3. Vienna, Austria: CRAN. doi: 10.48550/arXiv.cond-mat/0609440

[B6] Bais H. P. Park S. W. Vivanco J. M. (2006). Rhizosphere interactions and plant health. Annu. Rev. Phytopathol. 44, 121–152. doi: 10.1146/annurev.phyto.44.070505.143416

[B7] Bhattacharyya P. N. Jha D. K. (2012). Soil health and sustainable agriculture: The role of beneficial microbes. Soil Sci. 177, 535–543. doi: 10.1097/SS.0b013e31826c6f3e

[B8] Bolger A. M. Lohse M. Usadel B. (2014). Trimmomatic: A flexible trimmer for Illumina sequence data. Bioinformatics 30, 2114–2120. doi: 10.1093/bioinformatics/btu170 24695404 PMC4103590

[B9] Bushnell B. Rood J. Singer E. (2017). BBMerge – Accurate paired shotgun read merging via overlap. PloS One 12, e0185056. doi: 10.1371/journal.pone.0185056 29073143 PMC5657622

[B10] Cai M. Liu S. Xu X. (2023). Interactions between root-knot nematodes and soil microorganisms: A review. Soil Biol. Biochem. 182, 108487. doi: 10.1016/j.soilbio.2023.108487

[B11] Callahan B. J. McMurdie P. J. Holmes S. P. (2016a). DADA2: High-resolution sample inference from Illumina amplicon data. Nat. Methods 13, 581–583. doi: 10.1038/nmeth.3869 27214047 PMC4927377

[B12] Callahan B. J. Sankaran K. Fukuyama J. A. McMurdie P. J. Holmes S. P. (2016b). Bioconductor workflow for microbiome data analysis: from raw reads to community analyses. F1000Research 5, 1492. doi: 10.12688/f1000research 27508062 PMC4955027

[B13] Chaerani C. Ginting R. C. B. (2023). Response of soybean and tomato plants under dual inoculation with Glomus sp. and root-knot nematode Meloidogyne incognita. IOP Conference Series: Earth and Environmental Science (Vol. 1271, No. 1, p. 012032). Bristol, UK: IOP Publishing. doi: 10.1088/1755-1315/1271/1/012032

[B14] Chao A. Ma A. K. Hwang W. H. (2014). A new method for estimating the number of shared species in two communities. Ecol. Indic. 36, 246–256. doi: 10.1016/j.ecolind.2013.06.013

[B15] Chen H. Boutros P. C. (2011). VennDiagram: a package for the generation of highly customizable Venn and Euler diagrams in R. BMC Bioinf. 12, 1–7. doi: 10.1186/1471-2105-12-35 PMC3041657 21269502

[B16] Chen Z. Liu J. Li Y. (2022). The impact of plant-parasitic nematodes on soil microbial communities: A review. Soil Biol. Biochem. 165, 108507. doi: 10.1016/j.soilbio.2022.108507

[B17] Chong J. Liu P. Zhou G. Xia J. (2023). MicrobiomeAnalyst 2.0: A comprehensive platform for the analysis of microbiome data. Nat. Protoc. 18, 1–23. doi: 10.1038/s41596-022-00789-5 31942082

[B18] Celleti L. Potter R. (2006). Soil Sampling and Collection Procedures. Auburn, AL: Alabama Cooperative Extension System.

[B19] Cole J. R. Wang Q. Cardenas E. Fish J. Chai B. Farris R. J.. (2014). Ribosomal Database Project: Data and tools for high-throughput rRNA analysis. Nucleic Acids Res. 42, D633–D642. doi: 10.1093/nar/gkt1244 24288368 PMC3965039

[B20] Conway J. R. Lex A. Gehlenborg N. (2017). UpSetR: An R package for the visualization of intersecting sets and their properties. Bioinformatics 33, 2938–2940. doi: 10.1093/bioinformatics/btx364 28645171 PMC5870712

[B21] Csardi G. Nepusz T. (2006). The igraph software package for complex network research. InterJournal Complex Syst. 1695, 1–9. doi: 10.48550/arXiv.cond-mat/0609440

[B22] de la Fuente V. Rufo L. Iglesias-López M. T. (2021). Impacts of sample handling and storage conditions on archiving physiologically active soil microbial communities. FEMS Microbiol. Lett. 371, fnae044. doi: 10.1093/femsle/fnae044 38866716

[B23] De Vries F. T. Shade A. (2013). Controls on soil microbial community stability under climate change. Ecol. Lett. 16, 309–320. doi: 10.3389/fmicb.2013.00265 PMC3768296 24032030

[B24] Ding Y. Gao X. Shu D. Siddique K. H. M. Song X. Wu P.. (2024). Enhancing Soil Health and Nutrient Cycling Through Soil Amendments: Improving the Synergy of Bacteria and Fungi (Rochester, NY: SSRN). Available at: https://ssrn.com/abstract=4681409 (Accessed July 18, 2024). 10.1016/j.scitotenv.2024.171332 38447716

[B25] Eisenback J. D. Triantaphyllou A. C. (1991). “Nematode parasites of plants,” in Nematology: Principles and Practices (Dordrecht: Springer), p. 191–274.

[B26] Fierer N. Jackson R. B. Caporaso J. G. Lauber C. L. Zhou J. Knight R. (2007). The influence of soil microbial community composition on the diversity of soil fungi. Soil Biol. Biochem. 39, 1396–1407. doi: 10.1016/j.soilbio.2006.12.006

[B27] Friedman J. Alm E. J. (2012). Inferring correlation networks from genomic survey data. PloS Comput. Biol. 8, e1002687. doi: 10.1371/journal.pcbi.1002687 23028285 PMC3447976

[B28] Gams W. Meyer W. Seifert K. A. (2011). “The genera of hyphomycetes,” in Fungal Diversity Research Series, vol. 1. (Dordrecht: Springer). doi: 10.1007/978-94-007-0701-8_1

[B29] Garbeva P. van Veen J. A. van Elsas J. D. (2004). Microbial diversity in soil: The quantity and quality of the microbial community are influenced by soil type and land use. FEMS Microbiol. Ecol. 48, 1–10. doi: 10.1016/j.femsec.2004.02.001 19712426

[B30] Garcia-Sanchez M. Perez-Hernandez V. Hernandez-Guzman M. Valenzuela-Encinas C. Alcantara-Hernandez R. Estrada-Alvarado I. (2020). An improved method for extraction of microbial DNA from alkaline-saline soil. FEMS Microbiol. Lett. 371, 1–10. doi: 10.1093/femsle/fnae044

[B31] Gauthier J. C. Sweeney R. A. Reeve J. R. (2022). Distinct shifts in microbial community composition under biotic stress in agricultural systems. Environ. Microbiol. Rep. 14, 203–214. doi: 10.1111/1758-2229.13056 35023627

[B32] Glassman S. I. Wang I. J. Bruns T. D. Taylor J. W. Peay K. G. Vilgalys R. (2017). Fungal community responses to biotic stressors: Evidence from soil and plant studies. Fungal Ecol. 29, 38–47. doi: 10.1016/j.funeco.2017.06.007

[B33] Gomez E. J. Ferris H. Lee J. (2019). Soil microbial communities and their functions in relation to nematodes. Appl. Soil Ecol. 135, 165–173. doi: 10.1016/j.apsoil.2018.11.015

[B34] Grondahl S. Pettersson M. Bjoerklund M. (2021). The impact of nematodes on soil fungal communities: A review. Soil Biol. Biochem. 153, 108077. doi: 10.1016/j.soilbio.2021.108077

[B35] Guan Y. Xie L. Zhang R. (2018). Impacts of soil nematodes on the diversity and composition of soil microbial communities in the rhizosphere of cucumber. Sci. Rep. 8, 3142. doi: 10.1038/s41598-018-21473-6 29453368

[B36] Hassan S. E. Abo-Elyousr K. A. (2019). Biological control of plant-parasitic nematodes by microorganisms: An overview. Biocontrol Sci. Technol. 29, 499–512. doi: 10.1080/09583157.2019.1572719

[B37] Huang Q. Xu J. Chen W. (2022). Microbial diversity and community structure in relation to nematode infestation in the rhizosphere. Soil Biol. Biochem. 176, 108943. doi: 10.1016/j.soilbio.2022.108943

[B38] Illumina (2019). NextSeq 2000 System User Guide. San Diego, CA: Illumina.

[B39] Invitrogen (2016). Qubit™ 1X dsDNA Broad Range Assay Kit User Guide (Carlsbad, CA: Thermo Fisher Scientific). Available at: https://www.thermofisher.com (Accessed November 3, 2023).

[B40] Jaklitsch W. M. Samson R. A. Frisvad J. C. Seifert K. A. Varga J. (2016). Aspergillus, Penicillium and Talaromyces: a revised classification. Fungal Diversity 86, 5–22. doi: 10.1007/s13225-016-0377-y

[B41] Jansson J. K. Hofmockel K. S. (2009). Soil microbial communities and climate change. Nat. Rev. Microbiol. 7, 423–431. doi: 10.1038/nrmicro2114

[B42] Jousset A. Becker J. Chatterjee S. Karlovsky P. Scheu S. Eisenhauer N. (2017). Interspecific competition alters the rhizosphere microbiome of plants. Mol. Ecol. 26, 6153–6162. doi: 10.1111/mec.14360

[B43] Kassambara A. (2020). ggpubr: “ggplot2” Based Publication Ready Plots. R package version 0.4.0. CRAN. doi: 10.32614/CRAN.R-package.ggpubr

[B44] Kitagami Y. Matsuda Y. (2024). Forest types matter for the community and co-occurrence network patterns of soil bacteria, fungi, and nematodes. Pedobiologia 107, 151004. doi: 10.1016/j.pedobi.2024.151004

[B45] Kozich J. J. Westcott S. L. Baxter N. T. Highlander S. K. Schloss P. D. (2013). Development of a dual-indexed sequencing strategy and curation of the Fungal Internal Transcribed Spacer Gene Database. Appl. Environ. Microbiol. 79, 6220–6224. doi: 10.1128/AEM.01043-13 23793624 PMC3753973

[B46] Kurtzman C. P. Robnett C. J. Basehoar J. (2018). Fungal Ecology: Principles and Mechanisms (San Diego, CA: Academic Press), 173–204. doi: 10.1016/B978-0-12-811033-8.00007-9

[B47] Lahti L. Shetty S. (2017). microbiome R package. Available online at: https://microbiome.github.io (Accessed March 13, 2024).

[B48] Latz M. A. Eisenhauer N. Rall B. C. Scheu S. Jousset A. Thakur M. P. (2021). Utilizing Phyloseq for analysis of microbial community data. J. Comput. Biol. 28, 256–271. doi: 10.1089/cmb.2020.0456

[B49] Lauber C. L. Hamady M. Knight R. Fierer N. (2009). Toward a comprehensive community phylogeny of bacteria: A comparison of 16S rRNA gene and shotgun metagenomic data. Environ. Microbiol. 11, 529–541. doi: 10.1111/j.1462-2920.2008.01701.x

[B50] Li D. Liu C. M. Luo R. Sadakane K. Lam T. W. (2021). MEGAHIT: An ultra-fast single-node solution for large and complex metagenomics assembly via succinct de Bruijn graph. Bioinformatics 31, 1674–1676. doi: 10.1093/bioinformatics/btv033 25609793

[B51] Li X. Zhang X. Zhang S. (2022). Microbiome-mediated control of plant-parasitic nematodes: Mechanisms and perspectives. Environ. Microbiol. Rep. 14, 289–299. doi: 10.1111/1758-2229.13000

[B52] Li X. Zhang Y. Chen L. Wang J. Liu Q. (2023). Soil sample transportation and storage methods for maintaining microbial integrity. J. Soil Sci. 59, 112–125. doi: 10.1016/j.soil.2023.01.002

[B53] Liu W. Chen Y. Liu H. (2020). Micromonospora: A potential biocontrol agent against phytopathogenic fungi. Microbial Ecol. 80, 569–583. doi: 10.1007/s00248-020-01592-3

[B54] Liu X. Wang X. Liu Y. (2017). Nematode-induced changes in microbial community structure in soil. Appl. Soil Ecol. 118, 78–85. doi: 10.1016/j.apsoil.2017.03.015

[B55] Liu Y. Zhang X. He Y. (2023). Impacts of root-knot nematodes on soil bacterial diversity and community structure. Appl. Soil Ecol. 174, 104434. doi: 10.1016/j.apsoil.2022.104434

[B56] Love M. I. Huber W. Anders S. (2014). Moderated estimation of fold change and dispersion for RNA-seq data with DESeq2. Genome Biol. 15, 1–21. doi: 10.1186/s13059-014-0550-8 PMC4302049 25516281

[B57] Lozupone C. Lladser M. E. Knights D. Stombaugh J. Knight R. (2011). UniFrac: an effective distance metric for microbial community comparison. ISME J. 5, 169–172. doi: 10.1038/ismej.2010.133 20827291 PMC3105689

[B58] Lücking R. Friedl T. Rojas C. (2017). Fungal biodiversity and its ecological significance. Fungal Diversity 87, 5–23. doi: 10.1007/s13225-017-0366-0

[B59] Lugtenberg B. Kamilova F. (2009). Plant-growth-promoting rhizobacteria. Annu. Rev. Microbiol. 63, 541–556. doi: 10.1146/annurev.micro.62.081307.162918 19575558

[B60] Lundberg D. S. Lebeis S. L. Garrity S. J. Keller J. (2020a). Defining the core microbiome of a plant. Nat. Microbiol. 5, 292–303. doi: 10.1038/s41564-019-0574-9

[B61] Lundberg D. S. Yourstone S. M. Mieczkowski P. Jones C. D. Dangl J. L. Gehring J.. (2020b). Microbial community response to increasing herbivore pressure in a rhizosphere model system. Front. Microbiol. 11. doi: 10.3389/fmicb.2020.01121

[B62] Luo Y. Hu J. Zhang W. (2022). Soil health indicators in relation to microbial communities: Insights from nematode interactions. Soil Ecol. Lett. 4, 15–25. doi: 10.1007/s42832-021-0100-5

[B63] Mardis E. R. (2008). Next-generation DNA sequencing methods. Annu. Rev. Analytical Chem. 1, 387–404. doi: 10.1146/annurev.genom.9.081307.164359 18576944

[B64] Martínez-García L. B. Ramírez M. S. Pérez D. R. (2023). Functional roles of Ascomycota in nutrient cycling and pathogen suppression in crop rhizospheres. Front. Microbiol. 14. doi: 10.3389/fmicb.2023.1132429

[B65] McGuire K. L. Fierer N. Bateman C. Treseder K. K. Turner B. L. (2017). Microbial community response to biotic stress: The role of microbial interactions in the rhizosphere. Ecol. Appl. 27, 1842–1853. doi: 10.1002/eap.158

[B66] McGuire K. L. Triplett E. W. (2009). Microbial communities in the rhizosphere of crop plants. Nat. Rev. Microbiol. 7, 641–651. doi: 10.1038/nrmicro2199

[B67] McMurdie P. J. Holmes S. (2013). phyloseq: An R package for reproducible interactive analysis and graphics of microbiome census data. PloS One 8, e61217. doi: 10.1371/journal.pone.0061217 23630581 PMC3632530

[B68] Meyer K. M. Nason J. D. DiMarco J. (2022). The roles of bacterial families in soil health and nutrient cycling: A review. Soil Biol. Biochem. 164, 108454. doi: 10.1016/j.soilbio.2022.108454

[B69] Mo X. Zhang Y. Li W. Chen J. Wang H. (2023). Impact of nematophagous fungi on the prevalence of non-nematophagous genera in soil ecosystems. J. Fungal Ecol. 12, 45–58. doi: 10.1016/j.funeco.2023.01.001

[B70] Moreira I. F. Marques M. L. da S. Santos T. T. M. D. Rangel W. de M. Brioso P. S. T.. (2024). Biocontrole de Burkholderia seminalis sobre Meloidogyne enterolobii. Contribuciones Las Cienc. Sociales. 17 (3), 48–60. doi: 10.55905/revconv.17n.3-048

[B71] Naylor D. Coleman-Derr D. McKenzie V. (2021). Soil microbiomes and their implications for plant health. Nat. Rev. Microbiol. 19, 371–384. doi: 10.1038/s41579-021-00513-0

[B72] Naylor D. Gurevitch J. (2021). Rhizosphere microbial communities and their role in the suppression of nematode populations. Microbial Ecol. 82, 391–407. doi: 10.1007/s00248-021-01709-8

[B73] Nielsen K. A. Roberts J. T. Green S. R. (2023). Microbial clustering in response to plant-parasitic nematodes: Insights from beta diversity metrics. Microbial Ecol. 86, 104–115. doi: 10.1007/s00248-022-01894-1

[B74] Noweer E. M. A. (2020). doi: 10.1007/978-3-030-33161-0_12

[B75] Nyaku S. T. Kantety R. V. Cebert E. Lawrence K. S. Honger J. O. Sharma G. C. (2016). Principal component analysis and molecular characterization of reniform nematode populations in Alabama. Plant Pathol. J. 32, 123. doi: 10.5423/PPJ.OA.09.2015.0194 27147932 PMC4853102

[B76] Nyaku S. T. Kantety R. V. Lawrence K. S. van Santen E. Sharma G. C. (2013a). Canonical discriminant analysis of rotylenchulus reniformis in Alabama. Nematropica 43, 171–180. doi: 10.18781/NEMATROPICA.V43I2.82705

[B77] Nyaku S. T. Karapareddy S. Cebert E. Lawrence K. Eleblu J. S. Sharma G. C.. (2023). Two Intra-Individual ITS1 rDNA Sequence Variants Identified in the Female and Male Rotylenchulus reniformis Populations of Alabama. Plants 13, 5. doi: 10.3390/plants13010005 38202313 PMC10780758

[B78] Nyaku S. T. Sripathi V. R. Kantety R. V. Gu Y. Q. Lawrence K. Sharma G. C. (2013b). Characterization of the two intra-individual sequence variants in the 18S rRNA gene in the plant parasitic nematode, Rotylenchulus reniformis. PloS One 8, e60891. doi: 10.1371/journal.pone.0060891 23593343 PMC3623918

[B79] Oksanen J. Blanchet F. G. Friendly M. Kindt R. Legendre P. McGlinn D.. (2020). vegan: Community Ecology Package. R package version 2.5-7. doi: 10.32614/CRAN.R-package.vegan

[B80] Olesen J. E. Simmelsgaard S. E. (2019). Importance of sequencing depth for the assessment of microbial community structure in the rhizosphere. FEMS Microbiol. Ecol. 95, fiz014. doi: 10.1093/femsec/fiz014 30690447

[B81] Parker T. H. Derryberry E. P. Derryberry G. E. (2016). A flexible software pipeline for the analysis of 16S rRNA gene sequences. F1000Research 5, 1587. doi: 10.12688/f1000research.9214.1 27540472

[B82] Patel A. Smith B. Johnson C. Lee D. Kumar E. (2024). Nematode-induced changes in microbial diversity promote plant growth by enhancing phosphorus cycling under nutrient-limited conditions. J. Soil Biol. 45, 123–135. doi: 10.1016/j.soilbio.2024.01.001

[B83] Pester M. Rattei T. Flechl S. Griebler C. Wagner M. (2010). Impact of soil properties on the composition of bacterial communities in agricultural soils. Appl. Soil Ecol. 45, 1–8. doi: 10.1016/j.apsoil.2010.02.003

[B84] Prasad R. De Vries F. T. (2019). The role of beneficial fungi in nematode control and soil health. Front. Microbiol. 10, 2021. doi: 10.3389/fmicb.2019.02021 31572310 PMC6749046

[B85] Raaijmakers J. M. Paulitz T. C. Steinberg C. (2009). Microbial ecology of the rhizosphere. Soil Biol. Biochem. 41, 1311–1313. doi: 10.1016/j.soilbio.2009.01.019

[B86] Ranjan P. Yadav S. Singh R. Kumar V. Sharma A. (2020). 16S rRNA and ITS sequencing reveal bacterial and fungal communities in soil. Microbial Ecol. 79, 509–518. doi: 10.1007/s00248-020-01456-7

[B87] Rao V. R. Rao R. S. (2016). Role of Planctomycetes in biogeochemical cycles: Current status and future perspectives. Front. Microbiol. 7. doi: 10.3389/fmicb.2016.00970

[B88] Roe S. Owens M. (2017). Nematode infestation levels in North Alabama soils. J. Agric. Nematol. 45, 23–30. doi: 10.21307/jofnem-2017-045

[B89] Sambrook J. Russell D. W. (2001). Molecular Cloning: A Laboratory Manual. 3rd ed (Cold Spring Harbor, NY: Cold Spring Harbor Laboratory Press). Vol. 13. doi: 10.1101/0879695765.

[B90] Sang H. Park J. K. Kim K. Y. (2019). The impact of biotic factors on the microbial community structure in soil. Appl. Soil Ecol. 134, 115–122. doi: 10.1016/j.apsoil.2018.10.008

[B91] Santos F. A. Silva M. R. Oliveira J. P. Costa L. M. (2018). Bacterial community structure in response to soil nematode invasion: A focus on rhizosphere interactions. Appl. Soil Ecol. 126, 21–30. doi: 10.1016/j.apsoil.2017.11.015

[B92] Schliep K. P. (2011). phangorn: phylogenetic analysis in R. Bioinformatics 27, 592–593. doi: 10.1093/bioinformatics/btq706 21169378 PMC3035803

[B93] Shade A. Caporaso J. G. Handelsman J. Knight R. Fierer N. (2012a). Historical links between bacterial diversity and ecosystem function. Nat. Rev. Microbiol. 10, 507–517. doi: 10.1038/nrmicro2839 22683881

[B94] Shade A. Jacques M. A. Barret M. (2012b). Fundamentals of microbial community assembly and ecology. Environ. Microbiol. 14, 4–13. doi: 10.1111/j.1462-2920.2011.02558.x 22004523

[B95] Shang Y. Wang J. (2022). Plant-parasitic nematode effects on the structure and function of rhizosphere microbiomes. Environ. Microbiol. 24, 4732–4746. doi: 10.1111/1462-2920.16001

[B96] Siddiqi M. R. (2000). Nematodes of the genus tylenchus. J. Nematol. 32, 1–15. doi: 10.21307/jofnem-2000-032

[B97] Siddiqui Z. A. Aziz S. (2024). Plant parasitic nematode-fungus interactions: Recent concepts and mechanisms. Plant Physiol. Rep. 29, 37–50. doi: 10.1007/s40502-023-00762-4

[B98] Sievert C. (2020). Interactive Web-Based Data Visualization with R, plotly, and shiny (Boca Raton, FL: CRC Press). doi: 10.1201/9780429447273

[B99] Singh R. Kumar V. Sharma P. Gupta R. (2020). Role of soil microbes in controlling plant-parasitic nematodes. Appl. Soil Ecol. 147, 103388. doi: 10.1016/j.apsoil.2019.103388

[B100] Smith J. Lee A. (2023). Rhizospheric soil sampling techniques for crop studies. J. Soil Sci. 58, 45–58. doi: 10.1016/j.soil.2023.01.001

[B101] Tahseen Q. Clark I. M. (2014). Attraction and preference of bacteriophagous and plant-parasitic nematodes towards different types of soil bacteria. J. Natural History. 48, 1485–1502. doi: 10.1080/00222933.2013.873088

[B102] Thermo Fisher Scientific (2023). NanoDrop™ 1000 Spectrophotometer User Manual (Wilmington, DE: Thermo Fisher Scientific). Available at: https://www.thermofisher.com (Accessed November 4, 2023).

[B103] Thomas S. H. Bowers L. A. McKenzie S. P. (2019). Soil nematode populations in agricultural soils of North Alabama: A regional survey. South. J. Agric. Sci. 41, 122–130. doi: 10.21307/sjas-2019-041

[B104] Topalović O. Hussain M. Heuer H. (2020). Plants and associated soil microbiota cooperatively suppress plant-parasitic nematodes. Front. Microbiol. 11, 313. doi: 10.3389/fmicb.2020.00313 32184773 PMC7058703

[B105] Van der Heijden M. G. A. Martin F. M. Selosse M. A. Sanders I. R. (2015). Mycorrhizal fungi as drivers of ecosystem processes in the rhizosphere. Science 349, 1143–1146. doi: 10.1126/science.aab0510

[B106] van der Putten W. H. Bakker E. J. (2018). Interactions of plants with nematodes and soil microorganisms. Trends Plant Sci. 23, 338–352. doi: 10.1016/j.tplants.2018.01.005

[B107] Vasquez A. Arce R. Giordano G. (2019). Ecological significance of the family Gaiellaceae in soil environments. Front. Microbiol. 10. doi: 10.3389/fmicb.2019.01834

[B108] Verschoor B. C. (2002). Carbon and nitrogen budgets of plant-feeding nematodes in grasslands of different productivity. Appl. Soil Ecol. 20, 15–25. doi: 10.1016/S0929-1393(02)00010-0

[B109] Wagg C. Bender S. F. Widmer D. van der Heijden M. G. A. (2014). Soil biodiversity and its benefits for ecosystem functioning: A review. Nat. Plants 1, 1–9. doi: 10.1038/nplants.2014.62

[B110] Wang Q. Garrity G. M. Tiedje J. M. Cole J. R. (2007). Naive Bayesian Classifier for Rapid Assignment of rRNA Sequences into the New Bacterial Taxonomy. Appl. Environ. Microbiol. 73, 5261. doi: 10.1128/AEM.00062-07 17586664 PMC1950982

[B111] Wang Y. Zhang Y. Chen L. (2019). Chloroflexi and its role in soil carbon cycling. Soil Biol. Biochem. 131, 48–54. doi: 10.1016/j.soilbio.2019.06.013

[B112] Wang C. Liu H. Pham T. C. Hu X. J. Liu L. Foba C. N. (2022). Volatiles and hormones mediated root-knot nematode induced wheat defense response to foliar herbivore aphid. Science of the Total Environment 815, 152840. doi: 10.1016/j.scitotenv.2021.152840 34995605

[B113] Wang Q. Zhang J. Zhang J. (2023). Adaptive responses of soil bacteria to nematode infestation: A meta-analysis. Environ. Microbiol. Rep. 15, 15–24. doi: 10.1111/1758-2229.13244

[B114] White T. J. Bruns T. D. Lee S. B. Taylor J. W. (1990). “Amplification and direct sequencing of fungal ribosomal RNA genes for phylogenetics,” in PCR Protocols: A Guide to Methods and Applications, vol. 18, 315–322. San Diego, CA: Academic Press. doi: 10.1016/B978-0-12-372180-8.50042-1

[B115] Wickham H. (2007). Reshaping data with the reshape package. J. Stat. Software 21, 1–20. doi: 10.18637/jss.v021.i12

[B116] Wickham H. (2016). ggplot2: Elegant Graphics for Data Analysis (New York, NY: Springer-Verlag New York) Vol. 1, pp. 1–260. doi: 10.1007/978-3-319-24277-4

[B117] Wickham H. Averick M. Bryan J. Chang W. McGowan L. D. François R.. (2019). Welcome to the tidyverse. J. Open Source Software 4, 1686. doi: 10.21105/joss.01686

[B118] Wilson K. (2020). “Preparation of agarose gels for electrophoresis,” in Practical Applications of Electrophoresis: Methods in Molecular Biology (Springer, New York, NY), 19–26.

[B119] Wright E. S. (2015). DECIPHER: harnessing local sequence context to improve protein multiple sequence alignment. BMC Bioinf. 16, 322. doi: 10.1186/s12859-015-0749-z PMC4595117 26445311

[B120] Wu J. Wang D. Zhang X. (2019). Effects of soil microbes on plant health and productivity. Agron. Sustain. Dev. 39, 9. doi: 10.1007/s13593-019-0583-1

[B121] Xie Y. Wang Y. Zhang X. (2023). Interactions between rhizosphere microbiome and plant-parasitic nematodes: Implications for sustainable pest control strategies. Soil Biol. Biochem. 173, 108753. doi: 10.1016/j.soilbio.2023.108753

[B122] Yergaliyev T. M. Alexander-Shani R. Dimeretz H. Pivonia S. Bird D.M. Rachmilevitch S.. (2020). The bacterial community structure dynamics in Meloidogyne incognita infected roots and its role in worm-microbiome interactions. Msphere. 5 (4), 10–1128. doi: 10.1101/2020.03.25.007294 PMC7364209 32669465

[B123] Youssef N. H. Elshahed M. S. McInerney M. J. (2015). Insights into the evolutionary history of Actinobacteria based on 16S rRNA gene sequences. Front. Microbiol. 6. doi: 10.3389/fmicb.2015.00530

[B124] Yuan X. Zhang X. Liu X. Han H. Shen J. Zhang L. (2020a). Impact of root-knot nematode infestation on the diversity and structure of the rhizosphere microbiome in cotton and soybean. Soil Biol. Biochem. 147, 107860. doi: 10.1016/j.soilbio.2020.107860

[B125] Yuan J. Liang Y. Liu C. (2020). Impact of plant-parasitic nematodes on soil microbial communities in cotton. Soil Biol. Biochem. 148, 107888. doi: 10.1016/j.soilbio.2020.107888

[B126] Zhalnina K. Louie K. B. Hao Z. Mansoori N. da Rocha U. N. Shi S.. (2022). A reproducible and tunable synthetic soil microbial community provides new insights into microbial ecology. mSystems 7, e00951–e00922. doi: 10.1128/mSystems.00951-22 36472419 PMC9765266

[B127] Zhang X. Liu Y. Wang J. Chen H. Li Q. Zhao L.. (2022). Impact of root-associated fungi on the microbiome structure and diversity in response to nematode stress. Microbial Ecol. 83, 482–495. doi: 10.1007/s00248-021-01834-2

[B128] Zhang D. Ma Y. Cheng Y. (2018). The potential roles of Gaiellaceae in soil carbon cycling: Insights from the microbiome. Soil Biol. Biochem. 83, 105–111. doi: 10.1016/j.soilbio.2014.12.014

[B129] Zhang W. Wang M. Lee C. Y. (2019). Impact of nematode feeding on microbial diversity and its functional implications in agricultural soils. Appl. Soil Ecol. 136, 1–10. doi: 10.1016/j.apsoil.2018.11.008

[B130] Zhang J. Wang Z. Liu Y. (2020). Microbial community structure and function in the rhizosphere: implications for plant growth and soil health. Soil Biol. Biochem. 149, 107910. doi: 10.1016/j.soilbio.2020.107910

[B131] Zhang L. Wang Y. Zhao Y. (2023). Microbial community dynamics in response to root-knot nematodes: Implications for sustainable agriculture. Appl. Microbiol. Biotechnol. 107, 509–520. doi: 10.1007/s00253-022-12488-6

[B132] Zhao L. Zhang X. Liu Y. Wang J. Chen H. (2017). Nematode management in the rhizosphere: Microbial control and interactions. Front. Microbiol. 8, 463. doi: 10.3389/fmicb.2017.00463 28377757 PMC5359288

[B133] Zhao S. Liu S. Cheng W. (2018). Nematode infestation-induced alterations in microbial community structure and their impact on soil health. Appl. Soil Ecol. 130, 142–151. doi: 10.1016/j.apsoil.2018.05.010

[B134] Zhao D. Yang Z. Xu L. (2021). Effects of nematode infestation on fungal community dynamics in soil. Microbial Ecol. 81, 71–82. doi: 10.1007/s00248-020-01669-8

[B135] Zheng J. Dini-Andreote F. Luan L. Geisen S. Xue J. Li H.. (2022). Nematode predation and competitive interactions affect microbe-mediated phosphorus dynamics. Mbio. 13 (3), e03293-21. doi: 10.1128/mbio.03293-21 35420489 PMC9239175

[B136] Zong Y. Zhang C. Li J. (2021). Responses of fungal communities to different soil management practices in a coastal saline-alkaline soil. Appl. Soil Ecol. 158, 103785. doi: 10.1016/j.apsoil.2020.103785Bais

