# Supplementary figures and images for: Profiling of rhizosphere-associated microbial communities in North Alabama soils infested with varied levels of reniform nematodes
Source: Front Plant Sci. 2025 Mar 7;16:1521579. doi: 10.3389/fpls.2025.1521579 (PMC11925883; doi:10.3389/fpls.2025.1521579)

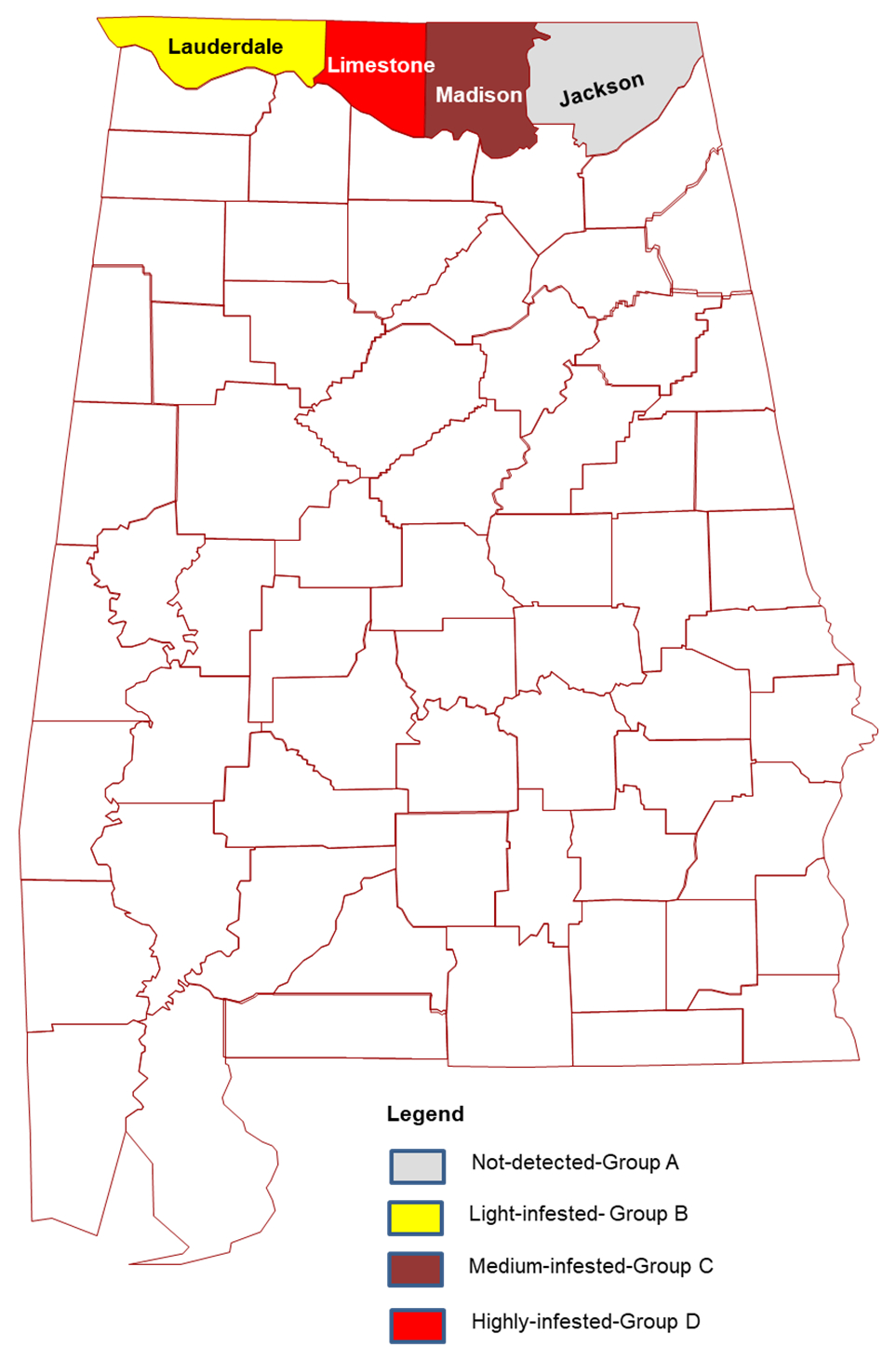

Supplement: Supplementary Figure 1 — North Alabama map showing levels of Reniform Nematode (RN) Infestation in four selected counties. [file Image1.jpeg]
